# Ultra-broadband and passive stabilization of ultrafast light sources by quantum light injection

**DOI:** 10.1515/nanoph-2024-0634

**Published:** 2025-04-24

**Authors:** Nicholas Rivera, Shiekh Zia Uddin, Jamison Sloan, Marin Soljačić

**Affiliations:** Department of Physics, 1812 Harvard University, Cambridge, MA 02138, USA; School of Applied and Engineering Physics, Cornell University, Ithaca, NY 14853, USA; Research Laboratory of Electronics, MIT, Cambridge, MA 02139, USA; Department of Physics, MIT, Cambridge, MA 02139, USA

**Keywords:** nonlinear optics, quantum optics, squeezed light, ultrafast optics

## Abstract

Nonlinear optical effects such as frequency conversion form the basis for many practical light sources. In a variety of settings, the performance of such sources is limited by quantum noise. In many nonlinear systems, this quantum noise gets strongly amplified, as a result of the large sensitivity of the nonlinear dynamics to changes in the initial conditions − a feature common to many nonlinear systems. Here, we develop a general theory of quantum noise resulting from nonlinear dynamics initiated by many-photon Gaussian quantum states. The theory provides guidelines to find the optimal quantum state to inject to maximally suppress the noise at the output. As a concrete example of the concept and theory, we consider the nonlinear optical phenomenon of supercontinuum generation by a femtosecond pulse, a famously noise-generating process, which is important in a range of applications in materials characterization and life science. By seeding supercontinuum generation with pulsed squeezed vacuum, one can achieve order-of-magnitude magnitude reduction of intensity and phase noise simultaneously, over a broad band of wavelengths, passively, and with no change in spectrum. The large magnitude and bandwidth of this effect is challenging to achieve by other means of stabilization, pointing to a promising approach for controlling quantum noise in a variety of nonlinear systems.

## 1 Introduction

Nonlinear optical effects have had tremendous implications for light sources with applications to sensing, imaging, medicine, and many other fields. In many settings, the performance of a nonlinear light source is limited by its noise in either intensity or phase. While extrinsic noise sources can often be mitigated, a fundamental noise source comes from the quantum vacuum fluctuations required by Heisenberg’s uncertainty principle. These quantum vacuum fluctuations can be seen as leading to noise in the initial conditions of the nonlinear optical process [1]. Many nonlinear systems display a strong sensitivity to initial conditions, leading to a strong amplification of these quantum vacuum fluctuations, creating strong and sometimes even macroscopic levels of noise at the output. The strong amplification of input noise has been observed in a wide range of phenomena including stimulated emission [2], Raman scattering [3], white-light or supercontinuum generation [4], superradiance by atomic ensembles [5], parametric oscillators [6], and free-electron lasers [7].

In this work, we propose a new and general route to countering the amplification of quantum noise in nonlinear systems. In particular, we show that by mixing the input of a nonlinear process with a properly chosen quantum state, such as a multimode squeezed state, one can strongly suppress the fluctuations of a wide variety of different outputs of the system. We start by providing a broad theoretical framework, called quantum sensitivity analysis, that enables one to predict what type of squeezed state leads to the greatest noise suppression, for any type of nonlinear interaction. As an example of the general idea, we then analyze a specific example of a noise-amplifying effect in nonlinear optics, which is supercontinuum or white-light generation. We choose this effect because it is an archetypical example of a strongly noise-amplifying process whose noise leads to limits in a variety of applications (such as materials characterization and biomedical imaging). We show that by mixing a femtosecond squeezed vacuum pulse with the input to conventional white-light generation, the intensity and phase fluctuations can both be reduced by an order of magnitude for realistic parameters. The effect described here is general and can be used to improve a wide range of nonlinear systems limited by noise (such as those mentioned in the first paragraph, as well as other laser systems such as fiber lasers and semiconductor lasers).

## 2 Results & discussion

**Figure 1:**
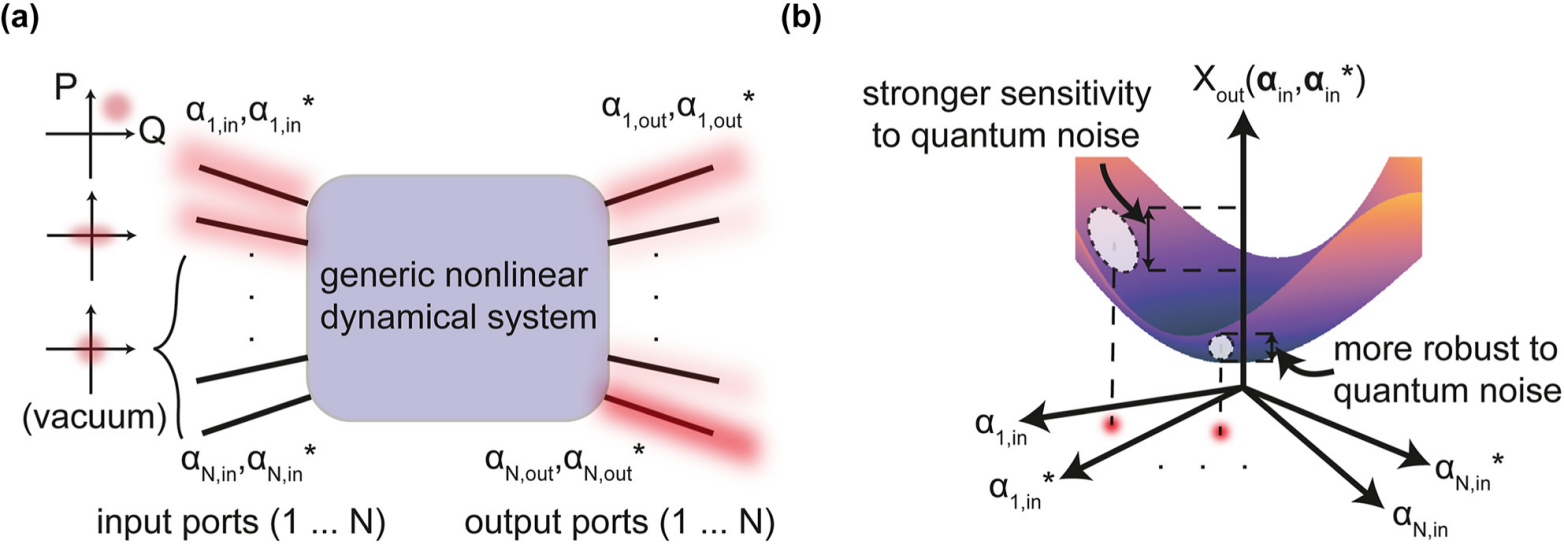
Quantum noise amplification as a result of classical sensitivity to initial conditions. (a) A generic nonlinear dynamical system can be specified as a map that connects inputs and outputs. These inputs can be complex wave amplitudes, particle positions and momenta, spins, etc. The inputs can be populated with excitations, or can be “dark,” indicating vacuum states for wave systems. The inputs undergo linear and nonlinear interactions represented by the purple box. These inputs have quantum fluctuations, schematically illustrated as phase-space distributions, though our theory also captures the effect of correlations between these inputs. Mixing a classical (coherent-state) input with quantum light (e.g., squeezed light or multimode entangled states) changes the quantum fluctuations at the input. (b) The crux of the framework is to analyze the sensitivity of observables *X*({*α*, α*}) to vacuum level shifts (quantum fluctuations) in the inputs *α*(0), α*(0). Larger (smaller) gradients translate to larger (smaller) noise in *X*.

We start by introducing the concept of noise amplification in multimode nonlinear systems. Although we will focus on nonlinear *optics* in the specific example, the physics we will describe applies to general nonlinear dynamical systems. Classically, a nonlinear system can be seen as a transformation of some input into an output. For nonlinear wave systems, we can resolve the input into a set of orthogonal modes with complex amplitudes $\left(\alpha_{i\text{,in}},\alpha_{i\text{,in}}^{*}\right)$ for the *i*th input mode (black lines in Figure 1a). The wave amplitudes *α*_*i*,in_ are normalized such that quantity $\alpha_{i\text{,in}}^{*}\alpha_{i\text{,in}}$ is the number of quanta in the *i*th mode (in the case of optics, the quanta are photons). In general, some inputs will have nonzero wave amplitude (red-filled colors in Figure 1a), and some will have zero amplitude. The nonlinear transformation in general leads to some distribution of amplitudes at the output $\left(\alpha_{i\text{,out}},\alpha_{i\text{,out}}^{*}\right)$ , which generally changes when the inputs (initial conditions) change [8].

Quantum mechanically, Heisenberg’s uncertainty principle enforces minimum fluctuations on the complex wave amplitudes, such that even when an input has zero mean amplitude, it has nonzero variance. This leads to noise at the outputs, since a fluctuation in the inputs maps to a fluctuation in the outputs through the nonlinear dynamics. The noise in the output will depend on two things: the noise and correlations of the input amplitudes, and the sensitivity of the outputs to changes in the inputs. Consider some quantity related to the output, denoted *X*_out_: it could be an intensity in some output mode, a phase, a product of amplitudes, etc. *X*_out_ is a function of the initial conditions: $X_{\text{out}}=X\left(\alpha_{\text{in}},\alpha_{\text{in}}^{*}\right)$ . Different initial conditions correspond to different values on the hypersurface of *X* shown in Figure 1b. When the initial conditions fluctuate, due to quantum noise, the resulting noise in *X* depends on the gradient of this hypersurface: larger gradients or equivalently, stronger sensitivity, translate to larger quantum noises. Therefore, in systems which display strong sensitivity to generic changes in initial conditions − a property shared by many nonlinear systems [9] − noise in most properties of the output becomes very strong due to strong amplification of the initial uncertainty.

We can make this intuition quantitative, via a universal connection between classical nonlinear dynamics and quantum noise, which we call the quantum mechanical law of total covariance, which was first derived in Refs. [10], [11], and which we review here. In this work, the new insight is related to how noise is controlled by initiating the dynamics with *quantum light* such as squeezed states and multimode entangled states, and its implications for ultrafast laser stabilization [12]. We provide a self-contained derivation of the quantum mechanical law of total covariance in the Supplementary Information (SI), where it is shown that the quantum noise in *X*_out_ can be determined in terms of derivatives of the classical input–output relation of Eq. (1), with respect to classical initial conditions: $\partial X_{\text{out}}/\partial \alpha_{\text{in}},\partial X_{\text{out}}/\partial \alpha_{\text{in}}^{*}$ . In particular, while the mean value of *X* is governed by the classical dynamics for some fixed initial conditions, the variance, ${\left(\Delta X_{\text{out}}\right)}^{2}$ is given by:

$${\left(\Delta X_{\text{out}}\right)}^{2}=v^{T}Cv,$$

where

(1) $$\begin{array}{ll} v & ={\left(\frac{\partial X_{\text{out}}}{\partial \alpha_{\text{in}}}\;\frac{\partial X_{\text{out}}}{\partial \alpha_{\text{in}}^{*}}\right)}^{T},\\ C & =\left(\begin{array}{cc} {〈\delta a\delta a〉}_{\text{in}} & {〈\delta a\delta a^{†}〉}_{\text{in}}\\ {〈\delta a^{†}\delta a〉}_{\text{in}} & {〈\delta a^{†}\delta a^{†}〉}_{\text{in}} \end{array}\right), \end{array}$$

where in the correlation matrix *C*, the operators *δa* ≡ *a* − ⟨*a*⟩, *δa* ^†^ ≡ *a* ^†^ − ⟨*a*⟩* refer to the noise creation and annihilation operators of the system modes and ⟨*δ* **a** *δ* **a**⟩_*ij*_ = ⟨*δa*_*i*_ *δa*_*j*_⟩ (with similar definitions for the other expectation values). The “in” subscript denotes that the correlation matrix is set by the statistics of the initial state, which allows for straightforward inclusion of the effects of excess noise (thermal or technical), multimode correlations (e.g., entanglement), and phase-sensitive correlations (e.g., from injecting squeezed states into the system at time zero). Similarly, a connected correlation function, of the form ⟨Δ*X*_out_Δ*Y*_out_⟩ = ⟨(*X* − ⟨*X*⟩)(*Y* − ⟨*Y*⟩)⟩, can be found as:

$$\left\langle \Delta X_{\text{out}}\Delta Y_{\text{out}}\right\rangle =v_{X}^{T}Cv_{Y},$$

where

(2) $$\begin{array}{ll} v_{X} & ={\left(\frac{\partial X_{\text{out}}}{\partial \alpha_{\text{in}}}\;\frac{\partial X_{\text{out}}}{\partial \alpha_{\text{in}}^{*}}\right)}^{T},\\ v_{Y} & ={\left(\frac{\partial Y_{\text{out}}}{\partial \alpha_{\text{in}}}\;\frac{\partial Y_{\text{out}}}{\partial \alpha_{\text{in}}^{*}}\right)}^{T}. \end{array}$$

with *C* defined as in Eq. (1).

This rule, we call the *quantum mechanical law of total covariance*, because it essentially prescribes a “quadrature-addition” rule for quantum noise − in parallel to the law of total variance in statistics, which shows that the variance of a sum of independent random variables adds in quadrature. Here, the same rule applies for quantum noise, where the relevant noises are the coefficients of *C* and they are weighted by the “sensitivities”: $\partial X_{\text{out}}/\partial \alpha_{\text{in}},\partial X_{\text{out}}/\partial \alpha_{\text{in}}^{*}$ . The quadrature-addition is especially clear in the simple but important case in which the initial fluctuations of all modes only have uncorrelated vacuum noise (meaning each input mode is in a coherent state). In that case:

(3) $$\begin{array}{ll} {\left(\Delta X_{\text{out}}\right)}^{2} & =\|\partial X_{\text{out}}/\partial \alpha_{\text{in}}{\|}^{2},\\ \left\langle \Delta X_{\text{out}}\Delta Y_{\text{out}}\right\rangle & ={\left(\partial X_{\text{out}}/\partial \alpha_{\text{in}}^{*}\right)}^{T}\left(\partial Y_{\text{out}}/\partial \alpha_{\text{in}}\right) \end{array}$$

and the quantum noise and correlations are *entirely* prescribed by the derivatives of the classical problem.

**Figure 2:**
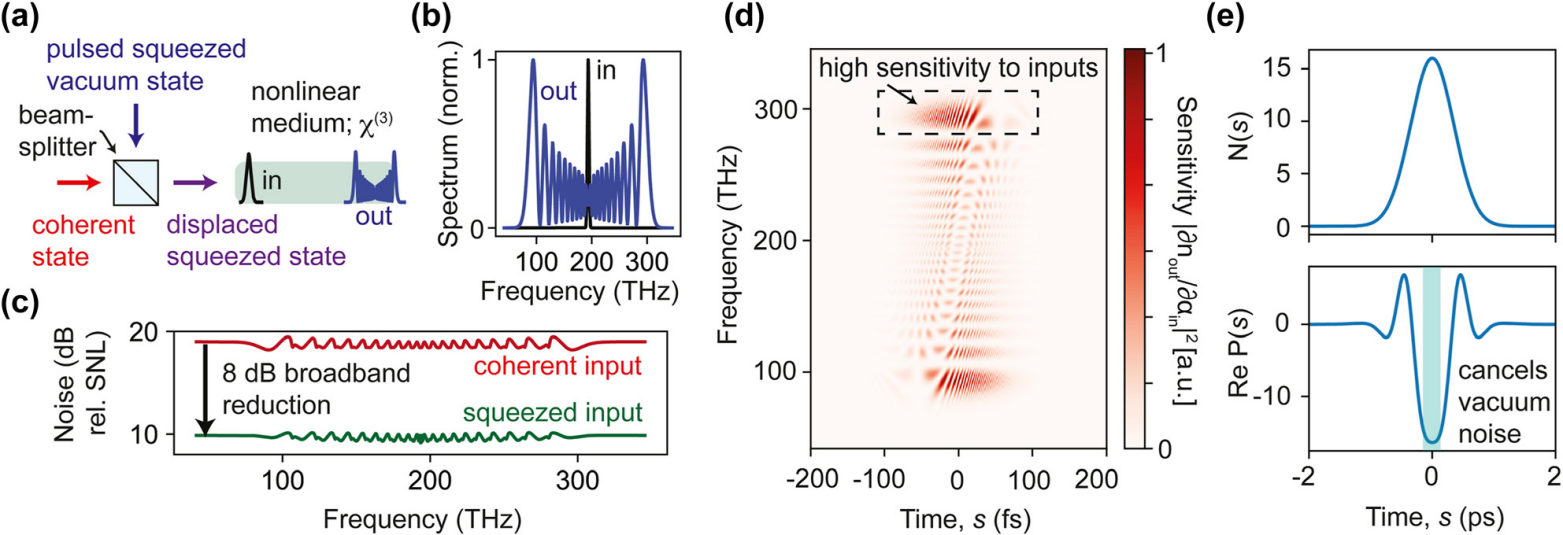
Driving ultrafast frequency conversion with squeezed femtosecond pulses. (a) Concept for lowering the noise of individual wavelengths generated in a frequency-broadening process. By seeding a frequency-broadening phenomenon (such as supercontinuum generation) with a displaced-squeezed pulse (realized by mixing a coherent state and squeezed state via a beamsplitter), the noise of the individual wavelengths can be strongly reduced. (b) Mean number of photons in each frequency after self-phase modulation of a Gaussian pulse. (c) Intensity noise (relative to shot noise) of individual wavelengths for coherent versus coherent-squeezed pulses of the same intensity, showing a nearly 10 dB broadband noise reduction of each wavelength. (d) Sensitivity of the spectral intensity to changes in the initial pulse amplitudes (in time-domain), |∂*n*_*ω*_/∂*α*(0, *s*)|^2^, and (e) the functions *N*(*s*) and *P*(*s*) referred to in the text. Parameters provided in SI.

**Figure 3:**
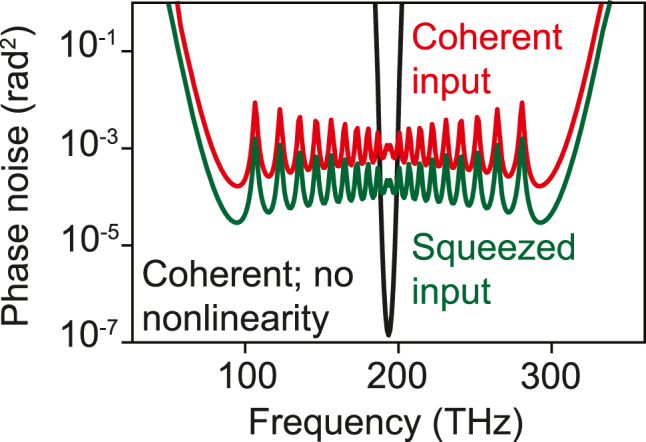
Broadband suppression of phase fluctuations by squeezed light injection. Spectral phase variance for a coherent pulse without nonlinearity (black) compared to the spectral phase variance for the same coherent pulse, showing strong amplification of phase noise beyond the coherent state limit. Injecting squeezed vacuum leads to broadband reduction in spectral phase noise (green). Parameters are the same as in Figure 2.

As an aside, we mention that while we have discussed noise in nonlinear bosonic systems described by creation and annihilation operators, this framework also applies to systems of particles. Although we will not need the result for the example we analyze in Figures 2 and 3, we state it to emphasize the generality of the framework. If one has a system specified in terms of a set of input positions **Q** and momenta **P** (e.g., a Newtonian system of particles subject to classical nonlinear dynamics), then a generic quantum correlation is found to be:

$$\left\langle \Delta X_{\text{out}}\Delta Y_{\text{out}}\right\rangle =v_{X}^{T}\tilde{C}v_{Y},$$

where

(4) $$\begin{array}{ll} v_{X} & ={\left(\frac{\partial X_{\text{out}}}{\partial Q_{\text{in}}}\;\frac{\partial X_{\text{out}}}{\partial P_{\text{in}}}\right)}^{T},\;v_{Y}={\left(\frac{\partial Y_{\text{out}}}{\partial Q_{\text{in}}}\;\frac{\partial Y_{\text{out}}}{\partial P_{\text{in}}}\right)}^{T},\\ \tilde{C} & =\left(\begin{array}{cc} {\left\langle \delta Q\delta Q\right\rangle }_{\text{in}} & \frac{1}{2}{\left\langle \delta Q\delta P+\delta P\delta Q\right\rangle }_{\text{in}}\\ \frac{1}{2}{\left\langle \delta Q\delta P+\delta P\delta Q\right\rangle }_{\text{in}} & {\left\langle \delta P\delta P\right\rangle }_{\text{in}} \end{array}\right). \end{array}$$

This framework for predicting noise based on classical dynamics can also readily be applied to coupled systems of waves and particles, as well as spin systems, as is relevant to effects such as spin-squeezing [13]. Further, in the SI, we show how many of the major noise models of quantum optics (e.g., of loss, gain, and second and third-order nonlinearity) can be straightforwardly derived using this approach − often much more simply than in the conventional quantum optical derivation based, e.g., on cumulant expansions [14]. Importantly, the result of this framework is that quantum noise can be calculated purely based on classical nonlinear simulations, by taking derivatives of the simulation results with respect to changes in the initial conditions. The relation of our theory to existing approaches such as cumulant expansions and phase-space methods is discussed in SI Section 1.3. Further, we note (see SI for more details) that the limit in which quantum noise is well-predicted by classical derivatives is the limit in which many photons are present in the initial state and many photons are needed to trigger nonlinearity, leading to Gaussian states at the output.

Using the intuition and framework discussed above, we now move to the main new results of this work: namely, we show how by seeding nonlinear systems with a judiciously chosen *squeezed state*, we can reduce the relevant fluctuations that get amplified by the dynamics, leading to much smaller noise at the output. This idea is conceptually related to the idea used to enhance the sensitivity of gravitational wave detection: there, by seeding an interferometer with squeezed light, the input vacuum fluctuations that normally limit the phase sensitivity can be reduced [15], [16]. Here, we show how by exploiting *multimode* squeezed light (e.g., femtosecond pulsed squeezed vacuum), we can realize a new and different application of quantum light: stabilizing complex light sources. In particular, we will consider nonlinear frequency conversion dynamics of ultrafast pulses propagating through third-order nonlinear media. Such frequency conversion processes − which are important from the standpoint of white-light generation − are famously noise-generating [17]. In particular, the different frequencies generated all tend to have intensity and phase noise much larger than the shot-noise level associated with a multimode coherent state (see Reference [18]). This added noise imposes limitations in cases where intense white-light is important, e.g., biomedical imaging and materials spectroscopy [19]. We will now show that by mixing an ultrafast squeezed vacuum pulse, with the input to these white-light–generating interactions, the noise in intensity (Figure 2) and phase (Figure 3) can be simultaneously lowered in all of the frequencies by over an order of magnitude with no change in the spectrum.

We consider the dynamics of an ultrafast pulse going through a medium with third-order nonlinearity, such as an optical fiber or waveguide, as illustrated in Figure 2a. We consider the case where dispersion can be neglected, for example, when the center wavelength of the pulse overlaps with the zero-dispersion wavelength of the optical fiber, and the pulse duration is sufficiently long. In this case, the frequency broadening comes from pure self-phase modulation, but a similar strategy should work for cases where other effects, such as dispersive-wave generation and stimulated Raman scattering, matter [4] (such as the case of supercontinuum generation pumped by light in the anomalous dispersion regime). The generalization to finite dispersion readily follows from Eq. (1). The classical description of this problem is an equation of motion for the envelope of the pulse, denoted *α*(*z*, *t*), which is a function of the length of propagation *z* and the time coordinate *t*, which measures the position along the pulse in a reference frame copropagating with the pulse. For a third-order nonlinear medium with negligible dispersion, the equation of motion is simply [20]:

(5) $$\partial_{z}\alpha \left(z,t\right)=i\gamma \alpha {\left(z,t\right)}^{*}\alpha {\left(z,t\right)}^{2},$$

where *γ* measures the nonlinear strength, and *α* is normalized such that |*α*|^2^(*z*, *t*) is the number of photons per unit time. The solution to this problem is simply $\alpha \left(L,t\right)={\text{e}}^{\text{i}\theta |\alpha \left(0,t\right){|}^{2}}\alpha \left(0,t\right)$ , with *θ* ≡ *γL* where *L* is the length of propagation. We are interested in the fluctuations of spectral quantities, *X*_*ω*_, in a range of frequencies with center *ω* and width Δ*ω*. *X*_*ω*_ could, for example, be the number of photons associated with frequency *ω*, or the spectral phase. According to the quantum mechanical law of total covariance, these fluctuations are given by

(6) $$\begin{array}{ll} {\left(\Delta X_{\omega }\right)}^{2} & =\int ds\;{\left|\frac{\partial X_{\omega }}{\partial \alpha \left(0,s\right)}\right|}^{2}\\ & \;+2\int dsds^{\prime }\;\left[\frac{\partial X_{\omega }}{\partial \alpha \left(0,s\right)}\frac{\partial X_{\omega }}{\partial \alpha^{*}\left(0,s^{\prime }\right)}\right]N\left(s,s^{\prime }\right)\\ & \;+2\int dsds^{\prime }\;\text{Re}\left[\frac{\partial X_{\omega }}{\partial \alpha \left(0,s\right)}\frac{\partial X_{\omega }}{\partial \alpha \left(0,s^{\prime }\right)}P\left(s,s^{\prime }\right)\right], \end{array}$$

where $N\left(s,s^{\prime }\right)\equiv \left\langle \delta a\left(0,s\right)\delta a^{†}\left(0,s^{\prime }\right)\right\rangle$ and $P\left(s,s^{\prime }\right)\equiv \left\langle \delta a\left(0,s\right)\delta a\left(0,s^{\prime }\right)\right\rangle$ represent phase-insensitive and phase-sensitive quantum correlations of the input pulse. For coherent-state inputs, which is the conventional case, the variance is given simply by the first term. In what follows, we will consider a simpler case where different time-slices *s* at the input are uncorrelated, such that $N\left(s,s^{\prime }\right)=N\left(s\right)\delta \left(s-s^{\prime }\right)$ , $P\left(s,s^{\prime }\right)=P\left(s\right)\delta \left(s-s^{\prime }\right)$ (expressions for *N* and *P* are given in the next paragraph). This uncorrelated structure can be realized in practice by nonlinear propagation in nondispersive nonlinear media. In what follows, we consider the spectral intensity $n_{\omega }=\alpha_{\omega }^{*}\alpha_{\omega }$ and spectral phase $\varphi_{\omega }=-i\log\left({\tilde{\alpha }}_{\omega }/|{\tilde{\alpha }}_{\omega }|\right)$ , where $\alpha_{\omega }\equiv \sqrt{\Delta \omega /2\pi }\alpha \left(L,\omega \right)=\sqrt{\Delta \omega /2\pi }\int_{-\infty }^{\infty }dt\;\alpha \left(L,t\right){\text{e}}^{\text{i}\omega t}$ and ${\tilde{\alpha }}_{\omega }\equiv \int_{-T/2}^{T/2}dt\;\alpha \left(L,t\right){\text{e}}^{\text{i}\omega t}$ (see SI for explanation of these definitions). The derivatives in Eq. (6) are given by:

(7) $$\begin{array}{ll} \frac{\partial n_{\omega }}{\partial \alpha \left(0,s\right)} & =|\alpha_{\omega }|\left({\text{e}}^{-\text{i}\varphi_{\omega }}\mu \left(\omega ,s\right)+{\text{e}}^{\text{i}\varphi_{\omega }}\nu^{*}\left(\omega ,s\right)\right),\\ \frac{\partial \varphi_{\omega }}{\partial \alpha \left(0,s\right)} & =\frac{\text{Sq}\left(s,T\right)}{2i|\alpha \left(L,\omega \right)|}\left({\text{e}}^{-\text{i}\varphi_{\omega }}\mu \left(\omega ,s\right)-{\text{e}}^{\text{i}\varphi_{\omega }}\nu^{*}\left(\omega ,s\right)\right) \end{array}$$

with $\mu \left(\omega ,s\right)=\left(1+i\theta |\alpha \left(0,s\right){|}^{2}\right){\text{e}}^{\text{i}\theta |\alpha \left(0,s\right){|}^{2}}$ , $\nu \left(\omega ,s\right)=i\theta \alpha^{2}\left(0,s\right){\text{e}}^{\text{i}\theta |\alpha \left(0,s\right){|}^{2}}$ , and Sq(*s*, *T*) = 1 if |*s*| < *T* and zero otherwise. The derivatives with respect to conjugate variables follow by conjugation of Eq. (7).

We will now show that by controlling the input fluctuations *N*(*s*) and *P*(*s*), it is possible to strongly lower the noise of all of the wavelengths, by nearly an order of magnitude. Consider the system in Figure 2a: a beamsplitter is illuminated with an intense coherent input on one end (left) and pulsed squeezed vacuum (top). The pulsed squeezed vacuum can be generated by sending a pulse through a nonlinear medium, as has been realized in early experiments on squeezing in fibers [21], [22], [23]. We consider the case where the squeezed pulse is generated using self-phase modulation of a pulse through a *second* fiber with nonlinear coefficient *γ*′ and length *ℓ* with an incident pulse of amplitude *β*(*s*). At the end of the text, we discuss other possibilities for generating the squeezing, such as second-order nonlinear media. It then follows that *N*(*s*) = |*ζ*(*s*)|^4^, $P\left(s\right)=\left(1+i|\zeta \left(s\right){|}^{2}\right)i\zeta {\left(s\right)}^{2}{\text{e}}^{2\text{i}|\zeta \left(s\right){|}^{2}}$ . Here, $\zeta \left(s\right)\equiv \sqrt{\chi }\beta \left(s\right)$ , with *χ* = *γ*′*ℓ*. Further, we note that *β*(*s*) = |*β*(*s*)|e^i*ϕ*(*s*)^, with *ϕ*(*s*) being the phase of each time-slice of the pulse incident into the squeezer (see SI). We also briefly note that this fiber needs to be part of a 50/50 Sagnac interferometer to produce squeezed vacuum, as opposed to bright squeezed vacuum: see page 17 of the SI, specifically the paragraph above Eq. (S60). Stated differently, Kerr nonlinearity is known to take a coherent state and produce a displaced squeezed state. The interferometer converts the displaced squeezed state into a vacuum squeezed state. The physics is derived in Ref. [20]. This second fiber, despite being described by the same equations as the frequency-generating fiber (albeit with weaker nonlinearity than the frequency-generating fiber), is needed to create squeezed vacuum, which is squeezed along the “right” quadrature to lower noise of spectral intensity and phase, as we will show in Figures 2 and 3.

In Figure 2b, we show the classical expectation for the spectrum of a Gaussian pulse (185 fs FWHM) undergoing self-phase modulation. The amplitude of the incident pulse (the coherent part, after the beamsplitter) is chosen such that the nonlinear phase shift of the peak of the pulse is 20*π*, leading to a large spectral broadening into an octave-spanning output. Relative to shot noise, the noise of the individual wavelengths generated is far above the shot-noise level (nearly 100-times), despite the individual wavelengths at the input being shot-noise limited (Figure 2c). On the other hand, when injecting a squeezed pulse into the upper port of the beamsplitter in Figure 2a, the noise of the individual wavelengths can be brought down by nearly an order of magnitude, and in a nearly wavelength-independent manner (green curve).

Using the quantum sensitivity analysis expression of Eq. (6), we can account for the large bandwidth of the effect (i.e., the wavelength-independent noise reduction). In the case where the nonlinear phase shift of the coherent pulse is large, i.e., *θ*|*α*(0, *s*)|^2^ ≫ 1, the quantity ∂*X*_*ω*_/∂*α*(0, *s*) (where *X*_*ω*_ is either intensity or phase) is approximately real if *α*(0, *s*) is real for all *s*, as it would be for a Gaussian pulse. Therefore, Eq. (6) becomes very well-approximated as $\int ds\;\left(2N\left(s\right)+2\text{Re }P\left(s\right)+1\right){\left|\frac{\partial X_{\omega }}{\partial \alpha \left(0,s\right)}\right|}^{2}$ . The squared derivative is shown in Figure 2d. From this approximation, one sees that since Re*P*(*s*) can be sufficiently negative, (2*N*(*s*) + 2Re*P*(*s*) + 1) can be below 1, therefore, reducing the fluctuations below what one gets from a coherent state input. Moreover, the effect is essentially independent of *ω*, as the factor (2*N*(*s*) + 2Re*P*(*s*) + 1) has no *ω* dependence. From this explanation, the effect can be understood as the result of reducing the relevant vacuum fluctuations, which seed the frequency-generation process.

Moreover, one sees that in this approximation, the squeezed vacuum pulse that leads to strong intensity noise reduction is *precisely* the same as the one that leads to strong phase noise reduction, since the quantity 2*N*(*s*) + 2Re*P*(*s*) + 1 is independent of the quantity *X*_*ω*_, meaning that both intensity and phase noise are simultaneously reducible without changing the spectrum. Although one might expect based that intensity noise would decrease at the expense of phase noise, and vice-versa, both noises are in excess of the coherent state values, and thus are simultaneously reducible. The phase noise, like the intensity noise, is roughly 20 dB above the value expected if each frequency at the output were in a coherent state with the same mean number of photons.

This is confirmed in Figure 3, where we show how femtosecond squeezed-vacuum seeding also suppresses phase fluctuations over the spectral bandwidth of the broadened pulse. Without any nonlinearity, the spectral phase variance is simply ${\left(\Delta \varphi_{\omega }\right)}^{2}=T/\left(4|\alpha_{\omega }{|}^{2}\right)$ , where *T* is the same as defined above Eq. (7). This is plotted as the black curve in Figure 3. For frequencies far away from the bulk of the spectrum, the relative phase fluctuations are high, as expected, since the number of photons at such frequencies is very small. With nonlinearity (same parameters as in Figure 2), the spectrum broadens, leading to lower phase fluctuations at the newly generated frequencies (red curve). Nevertheless, the phase noise is far above the vacuum level. Once the input is mixed with a squeezed vacuum pulse, the phase noise drops by nearly an order of magnitude in a nearly spectrally independent way, much like in Figure 2.

It may be surprising to see that both intensity and phase noise are both lowered by the injection of a displaced-squeezed vacuum state into the frequency-broadening fiber. This is an effect that occurs for large nonlinear phase shift accrued by the pump pulse, and therefore, an effect which occurs in the regime of large noise amplification. In this regime, quantum noise in only one quadrature determines the noise amplification, and so reducing noise in that quadrature leads to reduction of both amplitude and phase noise. This can be seen from the expression $\delta a_{\text{out}}\left(s\right)=\mu \left(s\right)\delta a_{\text{in}}\left(s\right)+\nu \left(s\right)\delta a_{\text{in}}^{†}\left(s\right)$ , where *δa*_in_(*s*), *δa*_out_(*s*) are fluctuation operators at the input (output) at time-slice *s*, and *μ*, *ν* are given as above. For large nonlinear phase shift, *μ* and *ν* have the same complex argument (it is approximately *π*/2). As a result, *δa*_out_ ∼ *δX*_in_, where $X_{\text{in}}=\delta a_{\text{in}}+\delta a_{\text{in}}^{†}$ is the “amplitude” quadrature [24]. The optimal squeezing phase for the squeezed vacuum which is “mixed” in is the phase which causes the squeezing to be along the amplitude quadrature for each time-slice.

## 3 Conclusions

What makes this mechanism of noise suppression unique is that it is (a) passive, requiring no explicit intensity modulation system [25] while acting on femtosecond pulses; (b) high-bandwidth, acting on all wavelengths in a pulse at once, in both intensity and phase; and (c) leads to no change in the spectrum. From the sensitivity analysis expression in Eq. (1), one can see that this effect is in principle very general, applying beyond femtosecond pulses in single-mode waveguides. Similar ideas could be applied to mode-locked lasers, amplifiers, multimode nonlinear systems, and all other sources where quantum noise gets strongly amplified to macroscopic or near-macroscopic levels. While such squeezed light injection can come from a squeezed light source based on fibers, as we have discussed here, it may also be viable to consider squeezed pulses generated by a second-order nonlinear system such as an optical parametric oscillator or amplifier. We leave it to future work to evaluate whether squeezed states generated via second order processes can generate squeezing with the right correlation structure to lower noise in ultrafast applications. Finally, we mention that it would also be interesting to consider the seeding of nonlinear processes with other quantum states of light, notably non-Gaussian states (of which examples include Schrodinger “cat,” Gottesman–Kitaev–Preskill, and Fock states).

Finally, we discuss experimental implementation of the above concept, particularly the generation and injection of the squeezed vacuum state. The primary limitations with generating the squeezed vacuum state we described relate to generating squeezing using pulses in media such as fibers and waveguides: these limitations are standard and have been demonstrated to be solved, such as mitigating excess noise in the laser that generates the squeezing, and working in chirp-free conditions near the zero-dispersion wavelength. After generating the squeezed vacuum pulses, one needs to avoid losses between the squeezed-light generator and the beam-splitter that mixes the squeezed light and the coherent state in Figure 2. For example, if one has 50 % loss between the squeezer and the beamsplitter, one can only take advantage of at most 3 dB of squeezing. This is a generic challenge for the use of squeezed light but can be mitigated by careful control of the downstream optics. Further, dispersion after the squeezer will lead to a more complicated squeezing structure (described by *P*(*s*, *s*′)) and suboptimal noise reduction performance. Finally, one has to make sure that the squeezing phase of the light can be stabilized and the temporal overlap of the squeezed and coherent pulse at the beamsplitter remains high.

## Supplementary Material

Supplementary Material Details
