# Supplementary material for: Ultra-broadband and passive stabilization of ultrafast light sources by quantum light injection
Source: Nanophotonics. 2025 Apr 24;14(11):1857–64. doi: 10.1515/nanoph-2024-0634 (PMC12133217; doi:10.1515/nanoph-2024-0634)
Supplement: Supplementary file 1 — Supplementary Material Details [file j_nanoph-2024-0634_suppl_001.pdf]

Supplementary Information for:
Ultra-broadband and passive stabilization of ultrafast light sources by quantum light injection

Nicholas Rivera[1,2,*], Shiekh Zia Uddin[3], Jamison Sloan[3], and Marin Soljačić[3,4]

[1] *Department of Physics, Harvard University, Cambridge, MA 02138, USA.*

[2] *School of Applied and Engineering Physics, Cornell University, Ithaca, NY 14853, USA.*

[3] *Research Laboratory of Electronics, MIT, Cambridge, MA 02139, USA.*

[4] *Department of Physics, MIT, Cambridge, MA 02139, USA.* *

*Electronic address: `nrivera@fas.harvard.edu`.

Contents

S1. The quantum optical law of total covariance and quantum sensitivity analysis 2

S1.1. Noise of operators which are linear in fluctuation operators 4

S1.2. Application to shot-noise limited inputs 6

S1.3. Case of an input-output theory specified by position and momenta 7

S1.4. Relation to existing approaches 8

S2. Applying the framework to textbook problems in quantum optics 9

S2.1. Quantum noise in linear dissipation 9

S2.2. Continuous dissipation 11

S2.3. Parametric amplification 12

S2.4. Parametric oscillator 14

S2.5. Linear amplifier 15

S2.6. Kerr squeezing 16

S2.7. Summary of this section 17

S3. Quantum noise in self-phase modulation seeded by quantum light 17

S3.1. Limiting cases 21

S3.2. Parameters for Figs. 2 and 3 22

S3.3. Validity of the linearized approximation 23

S3.4. Other structures for squeezing and optimal noise control 25

S3.5. Influence of temporal dispersion 26

References 27

S1. THE QUANTUM OPTICAL LAW OF TOTAL COVARIANCE AND QUANTUM SENSITIVITY ANALYSIS

In this note, we derive a general formulation of quantum noise problems in nonlinear optics, in the regime where the incident light has many photons in it. The derivation we present below applies to more than interacting systems of light: as will be seen, our derivations also apply to any systems of bosons, and thus also cover the case of light interacting with matter degrees of freedom. The net result of this new formulation is what we call the quantum optical law of total variance. It

allows us to express *any* quantum noise – in a strongly-interacting, multimode system, consisting of light and matter degrees of freedom – in terms of a sum of independent variances coming from the effect of vacuum fluctuations in each mode of quantized light and matter fields.

Consider a system of bosons described by a set of annihilation and creation operators $a_i, a_i^\dagger$ where i is a generalized index, labeling not only different modes for a given boson, but also different types of bosons. We'll denote the vector of operators as $\mathbf{a}, \mathbf{a}^\dagger$ for compactness. For example, in a case where light interacts with phonons, as well as atoms capable of absorbing the light, the boson operators may label photon modes, phonon modes, and the effective bosonic modes which describe an absorbing material.

The equation of motion for the operators can be written schematically as:

$$\begin{aligned}\dot{\mathbf{a}} &= F(\mathbf{a}, \mathbf{a}^\dagger) \\ \dot{\mathbf{a}}^\dagger &= F^\dagger(\mathbf{a}, \mathbf{a}^\dagger),\end{aligned} \tag{S1}$$

where F is a generic operator function that produces the right-hand side of the Heisenberg equations of motion corresponding to the system Hamiltonian.

In the case where the number of bosons in the initial state is large, the quantum dynamics can be well approximated by the lowest-order fluctuations on top of the mean-field dynamics. This "linearization" approximation proceeds by expressing the operators as

$$\mathbf{a} = \boldsymbol{\alpha} + \delta\mathbf{a}, \tag{S2}$$

where $\boldsymbol{\alpha} \equiv \langle \mathbf{a} \rangle$, plugging it into the Heisenberg equation, and neglecting terms of higher order than linear in $\delta\mathbf{a}$. The equation of motion for the mean fields can be written as

$$\dot{\boldsymbol{\alpha}} = F(\boldsymbol{\alpha}, \boldsymbol{\alpha}^*), \tag{S3}$$

where the c-number function $F(\boldsymbol{\alpha}, \boldsymbol{\alpha}^*)$ corresponds to replacing all operators in $F(\mathbf{a}, \mathbf{a}^\dagger)$ by c-numbers. These equations will be nothing other than the classical equations of motion for the system. Meanwhile, the equation of motion for the fluctuations can be expressed as

$$\delta\dot{a}_i = \sum_j \frac{\partial F_i}{\partial \alpha_j}\delta a_j + \sum_j \frac{\partial F_i}{\partial \alpha_j^*}\delta a_j^\dagger \tag{S4}$$

where, importantly, the derivatives are with respect to the classical (c-number) function. The equation of motion for the creation operators just follows from conjugation.

The solution to these equations can be expressed as a Bogoliubov transformation, as:

$$\delta a_i(t) = \sum_j \mu_{ij}\delta a_j(0) + \nu_{ij}\delta a_j^\dagger(0). \tag{S5}$$

In what follows, we will make a new connection, showing that the μ, ν are in fact derivatives of the classical equations of motion with respect to the initial conditions. To see this, let us consider when we take the classical equation of motion Eq. S3 and evaluate the change in the solution when the initial conditions are varied. Assuming that a variation in the initial conditions, $\delta\boldsymbol{\alpha}$ leads to a sufficiently small change in the output fields, we may linearize the equations, writing them as

$$\delta\dot{\alpha}_i = \sum_j \frac{\partial F_i}{\partial \alpha_j}\delta\alpha_j + \sum_j \frac{\partial F_i}{\partial \alpha_j^*}\delta\alpha_j^* \tag{S6}$$

This equation is in correspondence with Eq. S4, and its solution is the same, as Eq. S4 is a linear equation, and thus is solved identically to a classical equation. In particular, we may write:

$$\delta\alpha_i(t) = \sum_j \mu_{ij}\delta\alpha_j(0) + \nu_{ij}\delta\alpha_j^\dagger(0), \tag{S7}$$

where μ, ν are identical to the quantum-mechanical case. But, by construction, we may also write

$$\delta\alpha_i(t) = \sum_j \frac{\partial\alpha_i(t)}{\partial\alpha_j(0)}\delta\alpha_j(0) + \frac{\partial\alpha_i(t)}{\partial\alpha_j^*(0)}\delta\alpha_j^*(0), \tag{S8}$$

allowing us to identify

$$\mu_{ij} = \frac{\partial\alpha_i(t)}{\partial\alpha_j(0)}, \nu_{ij} = \frac{\partial\alpha_i(t)}{\partial\alpha_j^*(0)}, \tag{S9}$$

completing the proof.

S1.1. Noise of operators which are linear in fluctuation operators

We now use this result to connect the calculation of quantum noise in multimode systems of light and matter to adjoint methods for solving numerical differential equations. Consider fluctuations in an observable δX, given by:

$$\delta X = \sum_i c_i\delta a_i + d_i\delta a_i^\dagger. \tag{S10}$$

For example, this applies to a nonlinear operator such as the number operator n, to lowest order in the fluctuations. Then, it would be the case that $c_i = \partial n(\boldsymbol{\alpha}, \boldsymbol{\alpha}^*)/\partial\alpha_i$ and $d_i = \partial n(\boldsymbol{\alpha}, \boldsymbol{\alpha}^*)/\partial\alpha_i^*$

where all αs are output quantities. Note that this assumes that a higher-order term of the form $\delta a_i^\dagger \delta a_j$ is being neglected, which is valid as long as the number of photons in the mode is much larger than the fluctuations of that mode (see also Section 3.4) for more details.

In terms of the results of the previous section, we may connect to *input* quantities

$$\begin{aligned}\delta X(t) &= \sum_{ij} \left[c_i \frac{\partial \alpha_i(t)}{\partial \alpha_j(0)} + d_i \frac{\partial \alpha_i^*(t)}{\partial \alpha_j(0)} \right] \delta a_j(0) + \left[c_i \frac{\partial \alpha_i(t)}{\partial \alpha_j^*(0)} + d_i \frac{\partial \alpha_i^*(t)}{\partial \alpha_j^*(0)} \right] \delta a_j^\dagger(0) \\ &= \sum_j \frac{\partial X(t)}{\partial \alpha_j(0)} \delta a_j(0) + \frac{\partial X(t)}{\partial \alpha_j^*(0)} \delta a_j^\dagger(0) \\ &\equiv \left[\delta \boldsymbol{a} \cdot \frac{\partial}{\partial \boldsymbol{\alpha}} + \delta \boldsymbol{a}^\dagger \cdot \frac{\partial}{\partial \boldsymbol{\alpha}^*} \right] X(\boldsymbol{\alpha}, \boldsymbol{\alpha}^*) \end{aligned} \tag{S11}$$

In the last line, and in what follows throughout this Supplement, we will omit explicit indication of time-zero quantities. We also briefly note that in the main text, for intuitive ease, we have replaced the time-zero label by "in" and the time-t label by "out". Further, in the last line, we have introduced the notation $X(\boldsymbol{\alpha}, \boldsymbol{\alpha}^*)$, which refers to the value of the observable X, calculated *classically*, assuming some initial conditions $\boldsymbol{\alpha}, \boldsymbol{\alpha}^*$. While classically, the initial conditions and their conjugates would not be independent, here, we take them as independent, since they refer to fluctuations in a and $a^\dagger$, which quantum-mechanically have a nonvanishing commutator.

Since this expression applies to any observable, we may now write a compact relation for the variance in an arbitrary observable, in an arbitrary coupled system of light and matter degrees of freedom, in this linearization approximation. This relation is what we refer to as the quantum optical law of total variance. Within this approximation, the variance of X, $(\Delta X)^2 = \langle (\delta X)^2 \rangle$, is expressed as a quadratic form:

$$\begin{aligned}(\Delta X)^2 &= v^T C v, \text{where} \\ v &= \begin{pmatrix} \frac{\partial X}{\partial \boldsymbol{\alpha}} & \frac{\partial X}{\partial \boldsymbol{\alpha}^*} \end{pmatrix}^T \\ C &= \begin{pmatrix} \langle \delta \mathbf{a} \delta \mathbf{a} \rangle & \langle \delta \mathbf{a} \delta \mathbf{a}^\dagger \rangle \\ \langle \delta \mathbf{a}^\dagger \delta \mathbf{a} \rangle & \langle \delta \mathbf{a}^\dagger \delta \mathbf{a}^\dagger \rangle \end{pmatrix}\end{aligned} \tag{S12}$$

The correlation matrix C which multiplies the gradients v is constructed from the statistics of the initial field, which allows for straightforward inclusion of the effects of excess noise, multimode correlations (e.g., entanglement), and phase-sensitive correlations (e.g., from squeezed states of light). Mixed states such as thermal states can also be readily accommodated by this approach, using for example correlators: $\langle \delta a_i^\dagger \delta a_j \rangle = \delta_{ij} (e^{\beta \hbar \omega_i} - 1)^{-1}$, $\langle \delta a_i \delta a_j^\dagger \rangle =$

$\delta_{ij}\left(1+(e^{\beta\hbar\omega_i}-1)^{-1}\right)$, $\langle\delta a_i \delta a_j\rangle = 0$, and $\langle\delta a_i^\dagger \delta a_j^\dagger\rangle = 0$, where $\beta = 1/kT$ with k Boltzmann's constant and T temperature.

This reformulation shows that the noise is in fact largely understood in terms of the gradient of the classical transformation of the light with respect to the initial conditions. This enables one to predict and understand the dynamics of fluctuations and noise based on the classical understanding of nonlinear optical effects developed over recent years. That said, one key aspect of this framework that goes "beyond" many classical studies is that: in this framework, one must also study the sensitivity of the system to dark modes, as it is these which often limits the noise performance in the presence of interactions. We note that it is these first-derivatives which are encoding the linearization employed in the derivation of the quantum optical law of total covariance.

Before moving on, we discuss when we expect these results to be valid. First, we emphasize that Eqs. (S5) with μ, ν given by Eq. (S9) is the more general result, Eqs. (S11) and (S12) and the associated main text equations are consequences of considering an operator whose fluctuations are well-described as being linear in fluctuation operators. For the types of operators we consider in the main text, the linear approximation is excellent (see also Supplementary Section S3.3). The broader validity of Eqs. (S5) and (S9) stem from the fact that for problems where nonlinearities are triggered by many photons, the underlying Hamiltonian can be well-approximated by a quadratic Hamiltonian, leading to linear operator equations of motion for the fluctuations. Provided that fluctuations are not macroscopically amplified, this will coincide with the linearization procedure outlined in this section. Such a treatment is ubiquitous when considering squeezing in second- and third-order systems for parameters sufficiently far away from transitions associated with diverging fluctuations. There are some cases where the operator is not well-described by something linear in fluctuations: an example is presented in Section S2.3: the photon number fluctuations of squeezed vacuum. That said, the fluctuations are accurately captured by taking Eqs. (S5) and (S9) as the starting point and using the complete expression for the photon number, rather than a linearized one.

S1.2. Application to shot-noise limited inputs

In this section, we present a simple limiting case of Eq. S14 which provides a versatile formula for understanding quantum noise in systems with many-degrees of freedom. This special case will also more explicitly illustrate the physics of the quantum optical law of total variance.

We consider the limiting case in which the initial inputs have only vacuum noise (e.g. no excess noise and no phase-sensitive correlations). Cases with excess noise are treated later on in the Supplement, as well as in the main text. In that case, the only non-vanishing elements of the correlation matrix C are $\langle \delta a_i \delta a_j^\dagger \rangle = \delta_{ij}$. In that case, we may immediately write that the variance of any observable X is given as

$$(\Delta X)^2 = (\partial X/\partial \boldsymbol{\alpha})^\dagger (\partial X/\partial \boldsymbol{\alpha}) = \sum_k \left| \frac{\partial X}{\partial \alpha_k} \right|^2. \tag{S13}$$

In writing this, we have used the fact that X must be real-valued (as it is an observable), implying $\partial X/\partial \alpha_k^* = (\partial X/\partial \alpha_k)^*$ for any mode k.

This expression tells us that to understand the noise in X, we can ask how sensitive it is classically to a change in the initial conditions in mode k, where the typical magnitude of such a change is of order 1. Note that the α_k are in "photon units", so that $|\alpha|^2$ represents a number of photons. So, for a macroscopic initial occupation of some mode with n photons on average, $|\alpha| \sim \sqrt{n}$. If some observable is highly sensitive to changes of initial conditions in some mode, or even moderately sensitive but to many modes, one can expect X to be noisy. This is to say that observables which feature a high degree of connectivity will display also large noise. This can be understood from the fact that all of the contributions to the noise in X are non-negative. Importantly, since the contributions to the noise are all non-negative, the only way to have zero noise in the output is if all the derivatives vanish. This can be understood as a more general version of a phenomenon called *partition noise*, in which the splitting of some quantity into orthogonal channels leads to enhanced noise for those individual channels. Our framework not only makes these effects evident, but explains what happens when an in-principle infinite or continuous number of modes is involved. Of course, our framework can also deal with a wide variety of noise phenomena in addition to partition noise effects, as we will now illustrate with examples.

S1.3. Case of an input-output theory specified by position and momenta

In the derivation above, we considered an input-output theory specified in terms of complex amplitudes α, α^*. That said, the key physical idea, of looking at small variations of classical inputs, is general, and holds for other types of degrees of freedom a system might have. For example, if instead of a wave system, we have a system of particles whose degrees of freedom are position and momenta, then we can calculate noise by differentiating with respect to position and

momenta. In what follows, we derive the quantum mechanical law of total variance for the case of a single particle described by a position and momentum. The multi-particle generalization, as well as the generalization to systems with particle and wave degrees of freedom, is straightforward.

To derive the case of a position-momentum description, the simplest thing to do is to take the quantum mechanical law of total variance for a system described by complex amplitudes α, α^*, and make a variable transformation to position and momentum variables Q, P (one can convince themselves a particle system can be described by complex degrees of freedom α, α^* that are simple linear combinations of Q, P). Defining: $Q = (\alpha + \alpha^*)/2$ and $P = (\alpha - \alpha^*)/2i$, and using the chain rule to relate α, α^* derivatives to Q, P derivatives, one immediately gets:

$$
\begin{aligned}
(\Delta X)^2 &= v^T C v, \text{where} \\
v &= \left(\frac{\partial X}{\partial Q} \, \frac{\partial X}{\partial P} \right)^T \\
C &= \begin{pmatrix} \langle (\delta Q)^2 \rangle & \frac{1}{2}\langle \delta Q \delta P + \delta P \delta Q \rangle \\ \langle \frac{1}{2}\langle \delta Q \delta P + \delta P \delta Q \rangle & \langle (\delta P)^2 \rangle \end{pmatrix}.
\end{aligned}
\tag{S14}
$$

The generalization to multiple positions and momenta follows immediately as Eq. (3) in the main text.

S1.4. Relation to existing approaches

Our approach is a consequence of performing a cumulant expansion of the quantum dynamics and truncating the dynamics to quadratic order. Further, one assumes that fluctuations are weak relative to the mean fields.

In that sense, we could say that our approach is equivalent to a truncated cumulant (or cluster) expansion. At the same time, what is new about our approach is that it provides a new analytical result that predicts how the noise and squeezing at the output of a nonlinear system depends on the quantum statistics of the input (with those statistics encoded by the Gaussian correlation functions). This result is critical for practical applications, and was missing from the literature. Another new result of our approach is that we show that quantum noise effects (at the level of Gaussian statistics) can be predicted from classical models for a very general variety of systems including both waves and particles.

Our theory also enables a computationally more efficient way to compute quantum noise compared to direct linearization approaches and phase-space approaches, which we expect will make

our method the "preferred" way to approach many problems involving Gaussian quantum noise for complex multimode systems. In particular, typically quantum noise in nonlinear systems is computed either by linearization approaches, which involves solving a new differential equation every time the input noise distribution changes, unless one computes a Green's function for the differential equation, which is often infeasible for systems with large numbers of degrees of freedom (such as ultrafast systems described by a continuous-time field). Another approach is stochastic simulations which take the initial state of the classical nonlinear dynamics as being drawn from an ensemble of initial conditions where the noise is the same as the quantum noise. This requires often solving the differential equation thousands or more times until the results are statistically converged. In our approach, noise can be computed by the method of adjoints, and so a single observable's noise (e.g., intensity at some frequency) can be computed with only two simulations, and for any input noise distribution.

S2. APPLYING THE FRAMEWORK TO TEXTBOOK PROBLEMS IN QUANTUM OPTICS

In this section, we apply the framework to a few cases where the answers are widely known. We do this so that the interested reader can work through some simple analytical examples, convince themselves of the framework, and also see the variety of cases that can be dealt with. In some cases, it will be the case that this version of the derivation allows one to do the calculations more simply than the standard textbook derivation. It also enables a new decomposition, allowing identification of how different noise sources contribute.

S2.1. Quantum noise in linear dissipation

Consider a linear dissipative process which passes through a fraction η^2 of incident light. A consistent model of the linear dissipation treats the attenuation as a beamsplitting operation which transfers the attenuated energy to a second mode. Calling the input modes α, β, and denoting the outputs by α', β', we have

$$\begin{aligned}\alpha' &= -\eta\alpha + i\sqrt{1-\eta^2}\beta \\ \beta' &= i\sqrt{1-\eta^2}\alpha + \eta\beta.\end{aligned} \tag{S15}$$

Now, let's consider the transformation of an operator such as the quadrature operator

$$X' = \alpha' e^{i\theta} + \alpha'^* e^{-i\theta}, \tag{S16}$$

and evaluate the variance $(\Delta X)^2$, which probes for example quadrature squeezing. Using the derivatives $\partial X'/\partial\alpha == -re^{i\theta}, \partial X'/\partial\alpha^* = -re^{-i\theta}, \partial X'/\partial\beta = ite^{i\theta}, \partial X'/\partial\beta^* = -ite^{-i\theta}$, we have

$$\begin{aligned}
(\Delta X)^2 &= v^T C v, \text{where} \\
v &= (\partial X/\partial\alpha\ \partial X/\partial\beta\ \partial X/\partial\alpha^*\ \partial X/\partial\beta^*)^T \\
C &= \begin{pmatrix} \langle\delta\alpha\delta\alpha\rangle & \langle\delta\alpha\delta\beta\rangle & \langle\delta\alpha\delta\alpha^*\rangle & \langle\delta\alpha\delta\beta^*\rangle \\ \langle\delta\beta\delta\alpha\rangle & \langle\delta\beta\delta\beta\rangle & \langle\delta\beta\delta\alpha^*\rangle & \langle\delta\beta\delta\beta^*\rangle \\ \langle\delta\alpha^*\delta\alpha\rangle & \langle\delta\alpha^*\delta\beta\rangle & \langle\delta\alpha^*\delta\alpha^*\rangle & \langle\delta\alpha^*\delta\beta^*\rangle \\ \langle\delta\beta^*\delta\alpha\rangle & \langle\delta\beta^*\delta\beta\rangle & \langle\delta\beta^*\delta\alpha^*\rangle & \langle\delta\beta^*\delta\beta^*\rangle \end{pmatrix}.
\end{aligned} \tag{S17}$$

This can easily be shown to match the quantum calculation, using the operator transformations $a' = -\eta a + i\sqrt{1-\eta^2}b, b' = i\sqrt{1-\eta^2}a - \eta b.$

As a more explicit example, let us also consider the change in the photon number variance after propagation through a linear beamsplitter. The output intensity in for example, mode 1, is given as

$$n' = \alpha'^*\alpha' = (-\eta\alpha^* - i\sqrt{1-\eta^2}\beta^*)(\eta\alpha + i\sqrt{1-\eta^2}\beta). \tag{S18}$$

Let us consider the simple, but very common case, in which the input light in mode a doesn't have phase-sensitive correlations, and the light in mode b has no photons incident. Then, the output fluctuations, according to this formalism are

$$(\Delta n')^2 = \left|\frac{\partial n'}{\partial\alpha}\right|^2 \langle\delta\alpha\delta\alpha^* + \delta\alpha^*\delta\alpha\rangle + \left|\frac{\partial n'}{\partial\beta}\right|^2 \tag{S19}$$

Using $\partial n'/\partial\alpha = \eta^2\alpha^*$ and $\partial n'/\partial\beta = -i\eta\sqrt{1-\eta^2}\alpha^*$ (note that β is zero so the quadratic term doesn't contribute), we get

$$\begin{aligned}
(\Delta n')^2 &= \eta^4|\alpha|^2\langle\delta\alpha\delta\alpha^* + \delta\alpha^*\delta\alpha\rangle + \eta^2(1-\eta^2)|\alpha|^2 \\
&\equiv l^2(\Delta n)^2 + l(1-l)\langle n\rangle,
\end{aligned} \tag{S20}$$

where we have defined the transmission fraction $l = \eta^2$. This result is equivalent to the well-known formula which describes the change in intensity statistics due to dissipation (in more technical terms, this formula prescribes the noise figure of linear dissipation) [1].

S2.2. Continuous dissipation

In this section, we show that the framework also accounts for continuous processes of dissipation, where vacuum fluctuations are continuously coupled to the system. This example represents a significant extension in the scope of our framework. From the quantum optical law of total variance, we see that the noise depends on derivatives with respect to initial conditions. However, the equation of motion for a leaky cavity leads to vacuum fluctuations which can enter the cavity continuously, not just at the initial time, raising questions as to whether our framework could capture such cases. However, in the quantum mechanical theory of dissipation, it is known that these terms which describe continuously entering vacuum fluctuations (specifically, the term is a Langevin force) can be expressed in terms of the initial conditions of a reservoir of far-field modes. Therefore, the effect of continuous loss is treatable in our framework. We now demonstrate this.

Let us consider the classical description of a leaky cavity within temporal coupled mode theory [2]. For a cavity with one in-coupling port, we can write

$$\dot{a} = -\kappa a + \sqrt{2\kappa} b(t), \tag{S21}$$

where κ is the rate of decay of the intracavity amplitude. The field $a(t)$ is normalized such that $|a(t)|^2$ is the number of photons in the cavity. The term $b(t)$ is an injected input signal, normalized so that $|b(t)|^2$ is the number of photons per unit time incident on the cavity. For a cavity with no input, the term $b(t)$ is zero. However, in quantum calculations related to noise, we need to keep it: the essence of the quantum theory of noise presented in this work is that by treating $b(t)$ as a vacuum-fluctuating amplitude, the noise can be calculated by looking at derivatives with respect to the initial conditions. Said differently, while the field $b(t)$ might be zero on average, the derivative of the cavity field with respect to b is not zero. With that said, we calculate the evolution of the photon number noise with respect to time, due to cavity leakage.

The observable of interest is $a^*(t)a(t)$. The field $a(t)$ follows from Eq. S21 as

$$a(t) = a(0)e^{-\kappa t} + \sqrt{2\kappa} \int_0^t dt' e^{-k(t-t')} b(t'). \tag{S22}$$

The field $b(t)$ can be written in terms of independent degrees of freedom (which we will differentiate with respect to) as

$$b(t) = \int \frac{d\omega}{2\pi} e^{-i\omega t} b(\omega). \tag{S23}$$

Plugging this in to the solution for $a(t)$, we have

$$a(t) = a(0)e^{-\kappa t} + \sqrt{2\kappa}\int \frac{d\omega}{2\pi} b(\omega)\left[\frac{e^{-i\omega t} - e^{-\kappa t}}{\kappa - i\omega}\right]. \tag{S24}$$

The photon number is given by $n(t) = a^*(t)a(t)$ For an initial cavity state with excess phase-insensitive noise described by a Fano factor F, application of the quantum optical law of total variance yields

$$(\Delta n(t))^2 = \left|\frac{\partial n(t)}{\partial a(0)}\right|^2 F + \int \frac{d\omega}{2\pi}\left|\frac{\partial n(t)}{\partial b(\omega)}\right|^2. \tag{S25}$$

Recalling that the derivatives are taken with respect to the initial conditions $a(0) \neq 0$ and $b(\omega) = 0$, we have that

$$\left|\frac{\partial n(t)}{\partial a(0)}\right|^2 = e^{-4\kappa t} n(0) \tag{S26}$$

and

$$\left|\frac{\partial n(t)}{\partial b(\omega)}\right|^2 = e^{-2\kappa t} n(0)\frac{2\kappa}{\kappa^2 + \omega^2}(1 + e^{-2\kappa t} - 2\text{Re}\, e^{-i\omega t - \kappa t}). \tag{S27}$$

Evaluating the frequency integral in the quantum optical law of total variance yields

$$(\Delta n(t))^2 = l^2(\Delta n(0))^2 + l(1 - l)\bar{n}(0), \tag{S28}$$

where $l = e^{-2\kappa t}$ is the fractional intensity loss. This is precisely what is given from a quantum optical calculation.

S2.3. Parametric amplification

Here, we show how this framework readily allows one to consider parametric gain such as that provided by optical parametric amplifiers and oscillators which lead to squeezing. This shows immediately that our framework applies to second-order nonlinear effects.

For simplicity, we'll consider the case of a degenerate parametric amplifier: the non-degenerate case can also readily be considered. The equations of motion are [3]

$$\begin{aligned} \dot{a} &= \Omega a^* \\ \dot{a}^* &= \Omega^* a. \end{aligned} \tag{S29}$$

The solution is

$$\begin{pmatrix} a(t) \\ a^*(t) \end{pmatrix} = \exp\left[\begin{pmatrix} 0 & \Omega t \\ \Omega^* t & 0 \end{pmatrix}\right]\begin{pmatrix} a(0) \\ a^*(0) \end{pmatrix} = \begin{pmatrix} \cosh\left(|\Omega| t\right) a(0) + \sinh(|\Omega| t) e^{i\phi} a^*(0) \\ \sinh(|\Omega| t) e^{-i\phi} a(0) + \cosh\left(|\Omega| t\right) a^*(0) \end{pmatrix} \tag{S30}$$

where we have defined $\Omega = |\Omega| e^{i\phi}$. Let us now consider a quadrature operator $X_\varphi = a e^{i\varphi} + a^\dagger e^{-i\varphi}$. With this normalization, the quadrature variance in the vacuum state is one. Let us now ask about the squeezing of an initially injected vacuum state. From the quantum optical law of total variance, the quadrature variance is simply:

$$(\Delta X_\varphi)^2 = \left|\frac{\partial X_\varphi(t)}{\partial a(0)}\right|^2 = \left|\cosh\left(|\Omega| t\right) e^{i\varphi} + \sinh(|\Omega| t) e^{-i\phi} e^{-i\varphi}\right|^2. \tag{S31}$$

We briefly state a few limits of this formula. $\Omega = 0$ trivially returns $(\Delta X_\varphi)^2 = 1$. For a real pump phase, $\phi = 0$, we see that the amplitude quadrature variance ($\varphi = 0$) becomes $(\Delta X_0)^2 = e^{2\Omega t}$, while the phase quadrature variance becomes $(\Delta X_{\pi/2})^2 = e^{-2\Omega t}$. This is precisely the expected behavior. The conventional derivation of squeezing in a parametric amplifier looks similar up through Eq. 8 (where Eq.8 becomes an operator-valued equation coming from the Heisenberg equations). Then, one normally evaluates the quadrature operator by squaring X_φ. While slightly more algebraically involved, it is not much more so.

Let us highlight an important extension of the formula which is sigificantly more involved in the standard quantum derivation: the effect of excess noise. We'll show the case of phase-insensitive noise, but the phase-sensitive case readily derived as well. The influence of excess phase-sensitve noise is to have non-zero expectation values $\langle \delta a \delta a^\dagger + \delta a^\dagger \delta a \rangle$. This expectation value can readily be shown to be the Fano factor, F, for the incident light (the ratio of the intensity fluctuations to the mean intensity: for shot-noise limited light $F = 1$, while for sub- (super-) Poissonian light, $F < (>) 1$. In this case, the quantum optical law of total variance immediately implies

$$(\Delta X_\varphi)^2 = F\left|\frac{\partial X_\varphi(t)}{\partial a(0)}\right|^2 = F\left|\cosh\left(|\Omega| t\right) e^{i\varphi} + \sinh(|\Omega| t) e^{-i\phi} e^{-i\varphi}\right|^2. \tag{S32}$$

Finally, we emphasize that this case is one where the function $F = X$ vanishes at the argument it is evaluated at, but the derivative *does not*, leading to a finite noise at the output. Thus, even when the mean fields vanish, the fluctuations will still not in this framework. We should also mention however that for the intensity fluctuations, the function *and* its derivative vanish, incorrectly predicting vanishing fluctuations. This issue is however simple to fix. It is a result of considering an observable which is *nonlinear* in fluctuation operators, which violates the assumptions leading to Equations (S10) and (S11), where the fluctuations were linear in fluctuation operators. However, the fix is straightforward: compute the fluctuations in intensity retaining terms quadratic in fluctuation operators. For the case of squeezed vacuum generation, doing this can be shown to recover the known result that $(\Delta n)^2 = 2(\sinh^4 |\Omega| t + \sinh^2 |\Omega| t)$.

S2.4. Parametric oscillator

Here, we consider the example of a parametric oscillator: a cavity with a second-order nonlinear medium. The cavity is taken to have a partially transmitting mirror so that light can leak out, providing the useful squeezed beam.

The classical equation of motion for the system is simply that for a leaky cavity (as described by temporal coupled-mode theory) with terms containing parametric gain. In particular, we have

$$\dot{a} = -\kappa a + \Omega a^* + F(t), \tag{S33}$$

where $F(t)$ is a term which describes the change in the cavity field due to light sent into the cavity. Classically, if no light is sent in, the term can be dropped, but quantum mechnically we have to consider small non-zero values of the term coming from vacuum fluctuations. This will be manifested by looking at derivatives of the solution at time t with respect to $F(t')$ (for all t' between 0 and t). The in-coupling term $F(t) = \sqrt{2\kappa}b(t)$, where $b(t)$ is flux-normalized (bb^* has dimensions of photons per unit time). Quantum mechanically, $[b(t), b^\dagger(t')] = \delta(t-t')$. We can write the input field in a Fourier representation as $b(t) = \sum_\omega b_\omega(0)e^{-i\omega t}$, where $b_\omega(0)$ is the initial field of the input (time 0) at frequency ω.

The solution to Eq. S33 is

$$\begin{pmatrix} a(t) \\ a^*(t) \end{pmatrix} = S(t) \begin{pmatrix} a(0) \\ a^*(0) \end{pmatrix} + \sqrt{2\kappa} \int_0^t dt' S(t-t') \begin{pmatrix} b(t) \\ b^*(t) \end{pmatrix}, \tag{S34}$$

where

$$S(t) = \exp\left[\begin{pmatrix} -\kappa & \Omega \\ \Omega^* & -\kappa \end{pmatrix} t\right] = e^{-\kappa t} \begin{pmatrix} \cosh|\Omega|t & e^{i\phi}\sinh|\Omega|t \\ e^{-i\phi}\sinh|\Omega|t & \cosh|\Omega|t. \end{pmatrix} \tag{S35}$$

In the case of the optical parametric oscillator, we are interested in squeezing of the far-field beam emitted from the cavity. From classical temporal coupled mode theory, we know that the output field b_{out} is related to the input b by

$$b_{\text{out}}(t) = \sqrt{2\kappa}a(t) - b(t), \tag{S36}$$

where $b(t)$ is the input field and $a(t)$ is the intracavity field.

The observable of interest is the Fourier transformed quadrature of the output. In particular, if we mix the output field with a local oscillator (as is typical to do squeezing) – and consider balanced homodyne detection – the time-domain signature is the deviation of the current $\delta i(t)$

from the average value (which is set by the local oscillator). The time-domain current fluctuations are proportional to the quadrature operator $\delta i(t) \sim X_\varphi(t) = b_{\text{out}}(t)e^{i\varphi} + b^*_{\text{out}}(t)e^{-i\varphi}$. The phase φ is set by the relative phase of the local oscillator and the output beam, and is adjustable. Typically, the current is fed into an electrical spectrum analyzer, and we measure the power spectrum of the current $S(\omega) = |\delta i(\omega)|^2$. Note that in terms of the output fields, we have $\delta i(\omega) \sim \delta X_\phi(\omega) = b_{\text{out}}(\omega)e^{i\varphi} + (b_{\text{out}}(-\omega))^* e^{-i\varphi}$. By the input-output relation, we have: $b_{\text{out}}(\omega) = \sqrt{2\kappa}a(\omega) - b(\omega)$. For shot-noise limited inputs, the quantum optical law of total variance tells us that

$$|\delta X_\phi(\omega)|^2 = \left|\frac{\partial X_\phi}{\partial b(\omega)}\right|^2. \tag{S37}$$

We now calculate this derivative. First, we use the simplification that we're interested in *steady-state* squeezing: this means that we Fourier transform for some long but finite time-interval which is long after the oscillation dynamics start. Therefore, the transient terms associated with the initial conditions $a(0), a^*(0)$ can be neglected. After some algebra, one finds

$$a(\omega) = \sqrt{2\kappa}\frac{\kappa - i\omega}{(\kappa - i\omega)^2 - |\Omega|^2}b(\omega) + \sqrt{2\kappa}\frac{|\Omega|e^{i\phi}}{(\kappa - i\omega)^2 - |\Omega|^2}b(-\omega)^*. \tag{S38}$$

Using the input-output relation, one finds for the frequency-domain quadrature $\delta X_\phi(\omega)$:

$$\begin{aligned}\delta X_\phi(\omega) = &\left[\left(\frac{2\kappa(\kappa - i\omega)}{(\kappa - i\omega)^2 - |\Omega|^2} - 1\right)e^{i\varphi} + \left(\frac{2\kappa|\Omega|e^{i\phi}}{(\kappa - i\omega)^2 - |\Omega|^2}\right)e^{-i\varphi}\right]b(\omega)\\ &+ \left[\left(\frac{2\kappa(\kappa - i\omega)}{(\kappa - i\omega)^2 - |\Omega|^2} - 1\right)e^{-i\varphi} + \left(\frac{2\kappa|\Omega|e^{-i\phi}}{(\kappa - i\omega)^2 - |\Omega|^2}\right)e^{i\varphi}\right]b(-\omega)^*.\end{aligned} \tag{S39}$$

In what follows, we apply the quantum optical law of total variance to the most interesting case: $\phi = \pi$ and $\varphi = 0$. One gets

$$|\delta X_\varphi(\omega)|^2 = \frac{(\kappa - |\Omega|)^2 + \omega^2}{(\kappa + |\Omega|)^2 + \omega^2}, \tag{S40}$$

which at OPO threshold, gives zero at $\omega = 0$, leading to perfect squeezing (note that $\omega = 0$ corresponds to the cavity resonance frequency in the rotating frame which we wrote the equations in).

S2.5. Linear amplifier

In this section, we show that a linear amplifier can also be readily described with this framework. We will make use of the result derived in the section on continuous linear dissipation. In

fact, it turns out that: by taking $-\kappa \to G$ in the first term, and $\sqrt{2\kappa} \to \sqrt{2G}$ in the second term, one may repeat the derivation, arriving at the result that the intensity fluctuations of the output are

$$(\Delta n)^2 = g^2(\Delta n(0))^2 + g(g-1)\bar{n}(0), \tag{S41}$$

with $g = e^{2Gt}$, which agrees with the result in textbooks, e.g. [3]. In the shot-noise limited case, this leads to intensity fluctuations $g\bar{n}(0)(2g-1)$.

S2.6. Kerr squeezing

In this section, we show how to apply our theory to calculate quadrature squeezing in the Kerr effect. The standard framework derivation is presented in [4]. The equation of motion of a single mode of light undergoing self-phase modulation is

$$\dot{\alpha} = iK|\alpha|^2\alpha, \tag{S42}$$

whose solution is

$$\alpha(t) = e^{i\theta\alpha^*\alpha}\alpha, \tag{S43}$$

where $\alpha = \alpha(0)$ and $\theta = Kt$ is the nonlinear phase shift per photon. Evidently then, the intensity fluctuations follow simply as

$$(\Delta n)^2 = \left|\frac{\partial n}{\partial \alpha}\right|^2 = |\alpha|^2, \tag{S44}$$

where we have used $|\alpha(t)|^2 = |\alpha|^2$. More interesting are the fluctuations of the quadrature: $X_\varphi = \alpha(t)e^{i\varphi} + \alpha^*(t)e^{-i\varphi}$. This follows immediately from our theory as

$$(\Delta X_\varphi)^2 = \left|\frac{\partial X_\varphi}{\partial \alpha}\right|^2 = \left|(1 + i\theta|\alpha|^2)e^{2i\varphi+2i\theta|\alpha|^2} - i\theta\alpha^{*2}\right|^2. \tag{S45}$$

Related to this is the use of a Sagnac interferometer to produce squeezed vacuum using the Kerr effect. Without an interferometer, the pure Kerr effect leads to a bright (coherent) squeezed state. Since squeezed vacuum states are important for applications such as gravitational-wave interferometry, techniques to convert bright squeezed states into squeezed vacuum are important. The way to do this is to send light into a 50/50 Sagnac interferometer. Out of one port will come squeezed vacuum. The quadrature squeezing in that case can be readily derived. Passing the light through a 50/50 beamsplitter, followed by the Kerr effect, followed by another 50/50, leads to the following output field:

$$\alpha'(\alpha, \alpha^*, \beta, \beta^*) = -\frac{1}{2}e^{\frac{1}{2}i\theta(-\alpha^*-i\beta^*)(-\alpha+i\beta)}(-\alpha + i\beta) + \frac{i}{2}e^{\frac{1}{2}i\theta(-i\alpha^*-\beta^*)(i\alpha-\beta)}(i\alpha - \beta). \tag{S46}$$

The output noise is then

$$(\Delta X_\varphi)^2 = \left|\frac{\partial X_\varphi}{\partial \alpha}\right|^2 + \left|\frac{\partial X_\varphi}{\partial \beta}\right|^2 = \left|(1+\frac{1}{2}i\theta|\alpha|^2)e^{i(2\varphi+\theta|\alpha|^2)} - \frac{1}{2}i\theta\alpha^{*2}\right|^2, \qquad \text{(S47)}$$

which, one can show that the maximum squeezing is $4\Phi^2$, where Φ is the nonlinear phase shift of the light in the Kerr medium (it is half the phase-shift accumulated by the input, since the input gets split in half). Also notice that the squeezing is simply that of the fiber alone (with no beamsplitters), but evaluated at half the intensity (which makes sense since each nonlinear arm has half the input intensity after going through a 50/50 splitter).

S2.7. Summary of this section

To summarize this section, we have shown that a wide variety of standard results related to noise can be re-derived in our formalism. We have shown that discrete and continuous dissipative processes, parametric processes, and gain (an effect associated with matter degrees of freedom) can all be treated within our framework. This allows us to consider, with relative ease, arbitrary initial noise conditions, and phase-sensitive initial correlations as well. One may object that in the cases above, all of the problems correspond to quadratic Hamiltonians, where the Heisenberg equations can be mapped to equations for complex-numbers, making it clear that our framework should work. In what follows, we will consider cases now where the underlying dynamics are not quadratic (even though we will consider small fluctuations, which reduce the description to an effectively quadratic one).

In the next section, we apply the framework to several problems where the results are not known.

S3. QUANTUM NOISE IN SELF-PHASE MODULATION SEEDED BY QUANTUM LIGHT

In this section, we develop formulae describing the quantum noise and correlations induced by a many-mode process: namely, pure self-phase modulation (without dispersion) of a pulse in an instantaneous third-order nonlinear medium. Physically, this model approximates the quantum noise dynamics of a pulse propagating in a fiber or waveguide operated at its zero-dispersion wavelength. This problem can be analytically solved. Interestingly, although this case is analytical, the observables we will probe, namely single-wavelength noises, and their response to squeezed light injection, has not been examined before.

For a pulse in a medium experiencing pure self-phase modulation without temporal dispersion, the classical nonlinear equation of motion is

$$\partial_z \alpha(z,t) = i\gamma \alpha^*(z,t)\alpha^2(z,t), \tag{S48}$$

where $\alpha(z,t)$ is the envelope of the electric field of the pulse as a function of distance along the fiber z and time-delay relative to the center of the pulse (defined as time zero). Dimensions of α are chosen such that $|\alpha|^2 dt$ represents the number of photons carried in a time-span dt of the pulse. As is standard in ultrafast nonlinear optics, this equation is in the frame of reference which co-propagates with the pulse at its group velocity v_g [5]. The quantity γ is related to the nonlinear propagation phase.

In particular, the pulse envelope, after a propagation distance L in the fiber, is given by:

$$\alpha(L,t) = e^{i\theta\alpha^*(0,t)\alpha(0,t)}\alpha(0,t), \tag{S49}$$

where $\theta \equiv \gamma L$, which follows from the fact that $|\alpha|^2(z,t) = |\alpha|^2(0,t)$.

In what follows, we will be interested in the fluctuations in the intensity of different spectral components of the pulse (we will also be interested in phase fluctuations, but we present the intensity case for concreteness. The derivation for phase fluctuations follows essentially the same steps). This quantity is of direct interest for broadband white light sources, where nonlinearity is known to generate considerable noise. Classically, the spectrum is given by $n(L,\omega) = \alpha^*(L,\omega)\alpha(L,\omega)$, where ω is frequency, $n(L,\omega)$ is the spectral density of photons at frequency ω, and

$$\alpha(L,\omega) = \int dt\, e^{i\omega t}\alpha(L,t). \tag{S50}$$

Physically, to define a photon-number variance requires considering integrating the spectrum over some bandwidth $\Delta\omega$. The value of this bandwidth affects the measured noise, as is to be expected. In particular, if one integrates over the entire spectral bandwidth, then the number of photons out is equal to the number of photons in, and the fluctuations cannot change relative to the input. Similarly, if the bandwidth is infinitely narrow, there are few if any photons, and the statistics will degenerate to those of the vacuum state. Therefore, the quantity we consider is

$$n_\omega \equiv \int_{\omega-\Delta\omega/2}^{\omega+\Delta\omega/2} \frac{d\omega'}{2\pi} n(L,\omega'). \tag{S51}$$

In what follows, we will need to compute sensitivities such as $\partial n(L,\omega)/\partial\alpha(0,s)$, where $\alpha(0,s)$ is the complex amplitude of light at the entrance of the fiber at time slice s. To do this, we write

$$\frac{\partial n(L,\omega)}{\partial\alpha(0,s)} = \int dt_1 dt_2 e^{i\omega(t_2-t_1)} \left[\frac{\partial\alpha^*(L,t_1)}{\partial\alpha(0,s)}\alpha(L,t_2) + \alpha^*(L,t_1)\frac{\partial\alpha(L,t_2)}{\partial\alpha(0,s)}\right]. \tag{S52}$$

The relevant derivatives are given from the input-output relation as

$$\frac{\partial\alpha(L,t)}{\partial\alpha(0,s)} = \delta(t-s)\left(1+i\theta|\alpha(0,t)|^2\right)e^{i\theta|\alpha|^2(0,t)} \equiv \mu(s)\delta(t-s), \tag{S53}$$

and

$$\frac{\partial\alpha^*(L,t)}{\partial\alpha(0,s)} = \delta(t-s)\left(-i\theta\alpha(0,t)^{*2}\right)e^{-i\theta|\alpha|^2(0,t)} \equiv \nu^*(s)\delta(t-s), \tag{S54}$$

where we have defined μ and ν for ease of notation. Therefore:

$$\begin{aligned}\frac{\partial n(L,\omega)}{\partial\alpha(0,s)} &= \int dt_1 e^{i\omega(t_1-s)}\nu^*(s)\alpha(L,t_1) + \int dt_1 e^{i\omega(s-t_1)}\alpha^*(L,t_1)\mu(s)\\ &= e^{i\omega s}\mu(s)\alpha(L,\omega)^* + e^{-i\omega s}\nu^*(s)\alpha(L,\omega).\end{aligned} \tag{S55}$$

Using this, and the definition of the integrated spectrum n_ω, we can write that

$$\frac{\partial n_\omega}{\partial\alpha(0,s)} = \mu(s)\int_{\omega-\Delta\omega/2}^{\omega+\Delta\omega/2}\frac{d\omega'}{2\pi}e^{i\omega' s}\alpha(L,\omega')^* + \nu^*(s)\int_{\omega-\Delta\omega/2}^{\omega+\Delta\omega/2}\frac{d\omega'}{2\pi}e^{-i\omega' s}\alpha(L,\omega'). \tag{S56}$$

In what follows, we consider the case where the frequency window $\Delta\omega$ is sufficiently small such that $\alpha(L,\omega')\approx\alpha(L,\omega)$ over the integration range. In that case,

$$\frac{\partial n_\omega}{\partial\alpha(0,s)} = \frac{\Delta\omega}{2\pi}\text{sinc}\left(\frac{1}{2}\Delta\omega s\right)\left[\mu(s)\alpha(L,\omega)^* e^{i\omega s} + \nu^*(s)\alpha(L,\omega)e^{-i\omega s}\right]. \tag{S57}$$

From the quantum mechanical law of total covariance, derivatives like the one above "feed in" to the total variance $(\Delta n_\omega)^2$, via:

$$\begin{aligned}(\Delta n_\omega)^2 = \int dsds' &\left[\frac{\partial n_\omega}{\partial\alpha(0,s)}\frac{\partial n_\omega}{\partial\alpha^*(0,s')}\right]\langle\delta a(0,s)\delta a^\dagger(0,s') + \delta a^\dagger(0,s')\delta a(0,s)\rangle\\ &+ \left[\frac{\partial n_\omega}{\partial\alpha(0,s)}\frac{\partial n_\omega}{\partial\alpha(0,s')}\right]\langle\delta a(0,s)\delta a(0,s')\rangle + \left[\frac{\partial n_\omega}{\partial\alpha^*(0,s)}\frac{\partial n_\omega}{\partial\alpha^*(0,s')}\right]\langle\delta a^\dagger(0,s)\delta a^\dagger(0,s')\rangle.\end{aligned} \tag{S58}$$

For coherent state inputs, the result is simply:

$$(\Delta n_\omega)^2 = \int ds\left|\frac{\partial n_\omega}{\partial\alpha(0,s)}\right|^2. \tag{S59}$$

It is clear already from this expression that changing the quantum statistics of the input (as described by the various correlation functions) will lead to a change in the measured variance at the output. In what follows, we will consider what happens when the correlation functions correspond to a multimode squeezed input.

For concreteness, we will realize the multimode squeezed input as follows: take an second, auxiliary pulse, which is different from the one being used to generate the new frequencies. Send this auxiliary pulse through a Mach-Zehnder interferometer formed by two 50/50 beamsplitters with identical fibers in each arm (this may also be implemented in a Sagnac configuration). Assuming that this second pulse is at the zero-dispersion wavelength of this second fiber, the output will be a squeezed vacuum state, with correlations (see e.g. Ref. [4])

$$\begin{aligned}
\langle \delta b(s)\delta b^\dagger(s')\rangle &= |u(s)|^2\delta(s-s') \\
\langle \delta b^\dagger(s)\delta b(s')\rangle &= |v(s)|^2\delta(s-s') \\
\langle \delta b(s)\delta b(s')\rangle &= u(s)v(s)\delta(s-s') \\
\langle \delta b^\dagger(s)\delta b^\dagger(s')\rangle &= u^*(s)v^*(s)\delta(s-s'),
\end{aligned} \tag{S60}$$

where u, v are of the same form as μ, ν (respectively), but defined based on the nonlinear parameter Θ of the auxiliary fiber, and the input pulse envelope $\beta(s)$ of the auxiliary pulse. The nonlinear parameter Θ is given in terms of the parameters γ', ℓ in the main text by $\Theta = \gamma'\ell|\beta(0,s)|^2 = \chi|\beta(s)|^2 \equiv |\zeta(s)|^2$. By feeding this squeezed vacuum and the primary pulse into a beamsplitter that transmits primarily the squeezed vacuum and mostly attenuates the primary pulse, one can generate an output which has the mean value of the primary pulse (up to attenuation), and correlations given by the squeezed vacuum. We can therefore simply use the four b-correlation functions above as the ones to plug in for the expression for the total variance $(\Delta n_\omega)^2$. Doing so yields:

$$(\Delta n_\omega)^2 = \int ds\ (2N(s)+1)\Big|\frac{\partial n_\omega}{\partial \alpha(0,s)}\Big|^2 + 2\text{Re}\ \left[P(s)\left(\frac{\partial n_\omega}{\partial \alpha(0,s)}\right)^2\right], \tag{S61}$$

where $N(s) = |v(s)|^2$ and $P(s) = u(s)v(s)$. Identifying $u(s) = (1+i|\zeta(s)|^2)e^{i\zeta(s)}$ and $v(s) = i\zeta(s)^2e^{i\zeta(s)}$, we arrive as $N(s) = |u(s)|^2 + |v(s)|^2 - 1 = |\zeta(s)|^4$ and $P(s) = i\zeta(s)^2(1+i|\zeta(s)|^2)e^{2i\zeta(s)}$ as in the main text.

To conclude this section, we mention that the derivation of phase fluctuations follows identically to the derivation of intensity fluctuations, simply making use of the definition of the spectral phase $\phi(L,\omega)$ given in the main text. The derivation, following identically, leads to the general

Eq. (6) mentioned in the main text. A subtle point is related to the regularization of phase noise. In particular, for phase fluctuations, in defining a spectral amplitude which could be measured by a spectrometer, a relevant parameter is the duration of time that the spectrometer collects the field. This defines a timespan T, which could be seen as the window of a Fourier transform. The phase uncertainty is proportional to this parameter, simply because the longer T is, the more random phase kicks the field is subject to (due to vacuum fluctuations). Since there is no restoring force on the phase, the phase will walk-off indefinitely when subject to random kicks. Despite this, the T-dependence of all phase quantities we consider (either seeded by coherent or squeezed states), and with or without nonlinearity, has the same linear dependence on T. Thus, T merely sets an overall scale for the phase fluctuations..

S3.1. Limiting cases

Linear propagation. As a sanity check, note that in the absence of nonlinearity $\theta = 0$, we have $\mu(s) = 1$ and $\nu(s) = 0$. In this case, there are no dynamics, and we have

$$\frac{\partial n_\omega}{\partial \alpha(0,s)} = \int_{\omega-\Delta\omega/2}^{\omega+\Delta\omega/2} \frac{d\omega'}{2\pi} e^{i\omega' s}\alpha(0,\omega')^* = \frac{\partial n_{\omega,\text{in}}}{\partial \alpha(0,s)}, \tag{S62}$$

where the subscript "in" denotes a quantity evaluated after zero length of propagation (i.e., evaluated at the input). It follows immediately from the quantum mechanical law of total covariance that

$$(\Delta n_\omega)^2 = v^T C v = (\Delta n_{\omega,\text{in}})^2, \tag{S63}$$

where $v = \left(\frac{\partial n_{\omega,\text{in}}}{\partial \boldsymbol{\alpha}_\text{in}} \frac{\partial n_{\omega,\text{in}}}{\partial \boldsymbol{\alpha}^*_\text{in}}\right)^T$ and C is the initial correlation matrix at the input.

Statistics of the intensity integrated over the full spectrum. Noting that the total photon number $n_\text{out} = \int \frac{d\omega}{2\pi} n(L,\omega) = n_\text{in}$, it is immediately clear that the output variance has to equal the input variance. That said, in order to sanity check the specific algebraic steps, we show that the expressions, at least assuming coherent-state initial statistics, also reproduce this answer. Integrating over the full spectrum yields

$$\frac{\partial n_\omega}{\partial \alpha(0,s)} = \mu(s)\alpha(L,s)^* + \nu^*(s)\alpha(L,s). \tag{S64}$$

For input coherent-state statistics, the total noise is given by

$$(\Delta n_\omega)^2 = \int ds \left|\frac{\partial n_\omega}{\partial \alpha(0,s)}\right|^2 = \int ds (|\mu(s)|^2 + |\nu(s)|^2)|\alpha(L,s)|^2 + 2\text{Re}\left[\mu(s)\nu(s)(\alpha^*(L,s))^2\right]. \tag{S65}$$

Using $\alpha(L,t) = e^{i\theta\alpha^*(0,t)\alpha(0,t)}\alpha(0,t)$ and the definitions of $\mu(s)$ and $\nu(s)$, it is found that

$$(\Delta n_\omega)^2 = (\Delta n_{\text{out}})^2 = \int ds\, |\alpha(L,s)|^2 = n_{\text{out}}, \tag{S66}$$

indicating indeed that the full pulse on the output is in a coherent state.

S3.2. Parameters for Figs. 2 and 3

We collect relevant parameters for the calculations presented in Figs. 2 and 3. For the computation of the spectrum and intensity noise, the relevant parameters are the nonlinear phase $\theta|\alpha(0,s)|^2$ and the pulse duration σ. The absolute amplitude of the pulse and length of propagation do not individually appear in the output amplitude in the zero-dispersion regime considered in Eq. (5) of the main text. The peak nonlinear phase (at the pulse center) is taken to be 20π for both figures, to give a multi-octave spanning spectrum and noise amplification comparable to supercontinuum generation in the normal dispersion regime. The pulse is taken as an unchirped Gaussian of the form $e^{-t^2/\sigma^2+i\phi}$ with $\phi = 0$ and $\sigma = 78.6$ fs. For the intensity fluctuations, we defined a bandwidth $\Delta\omega$ over which the spectrum is defined (to have a finite number of photons in each spectral bin). The width taken is 1.01 THz in linear frequency, much smaller than the frequency-width of the broadened pulse. For the spectral phase fluctuations, the only change to the relevant parameters are the replacement of a spectral bandwidth $\Delta\omega$ with a temporal-collection window T. The temporal collection window is taken to be 200 ps, somewhat arbitrarily insofar as it is longer than the pulse duration.

For the squeezer pulse, the relevant parameters are the nonlinear phase $\zeta(s)$ the pulse duration, and the initial phase (analogous to ϕ in the previous paragraph). These together fully specify $N(s)$ and $P(s)$. The value of $\zeta(s)$ is taken to be 4 rad, $\sigma = 990$ fs, and the phase $\phi = -0.22\pi$. This phase choice leads to optimal noise reduction for intensity and phase. The peak squeezing (i.e., in the time-slice basis, at the peak of the pulse) is about 16 dB. The dependence of an observable, such as the spectral density at some frequency, has an oscillatory dependence on the phase ϕ as shown in Figure S1. The optimal phase depends on the number of photons at the input.

It may also be of interest to mention the displacement of the pulses considered. Regarding the displacement, because we are talking about pulses and thus continuous time creation and annihilation operators (which are delta-function normalized), we need to specify a temporal duration to talk about a dimensionless displacement. In particular, given a number of photons $\langle a^\dagger(s)a(s)\rangle\Delta s$

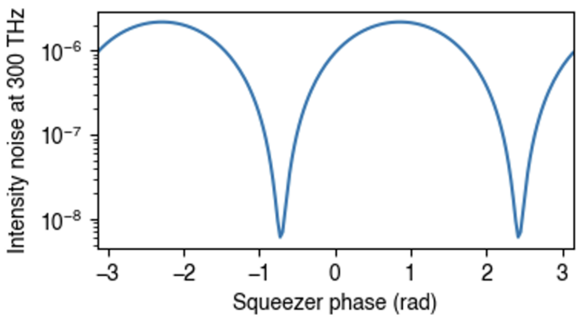

FIG. S1: Dependence on the squeezer phase ϕ of the spectral density at 300 THz after frequency broadening (for the same parameters in the main text).

in a time-window Δs, we can specify a displacement (in absolute value) as the square root of this number. This gives a sense of how far from the origin the states we are talking about are. For the pulse prior to frequency-broadening, if we consider a 1 fs window around the pulse peak, and a pulse with 10^8 photons per pulse (as we consider in the main text), the displacement in that time slice is of magnitude 10^3.

S3.3. Validity of the linearized approximation

In this section, we discuss the validity of the linearized approximation which leads to the quantum sensitivity analysis expressions. The linearization approximation entails a lack of coupling of higher-order moments to lower-order ones. In other words, the mean evolves without any influence from quadratic correlations and higher-order correlations. The quadratic correlators (the elements of the covariance matrix) are driven by the mean, but have no influence from third- and higher-order correlations, In what follows, we will evaluate the first correction to the mean number of photons and show that for the parameters we consider in this work, the magnitude of the correction from higher-order correlations is expected to be much smaller than the result from linearization. We will then argue that the corrections to the variances are of the same relative magnitude.

We consider the number of photons in a bandwidth $2\Delta\omega$:

$$n_{\omega,\Delta\omega} = \int_{\omega-\Delta\omega}^{\omega+\Delta\omega} \frac{d\omega}{2\pi} \langle a^\dagger(L,\omega) a(L,\omega) \rangle. \tag{S67}$$

This can be written in terms of the time-domain operators as

$$n_{\omega,\Delta\omega} = \frac{\Delta\omega}{\pi} \int ds ds' e^{-i\omega(s-s')} \text{sinc}\left(\Delta\omega(s-s')\right) \langle a^\dagger(L,s) a(L,s')\rangle. \tag{S68}$$

To evaluate this, we use the linearized relationship $a(L,s) = \alpha(L,s) + \mu(s)\delta a(0,s) + \nu(s)\delta a^\dagger(0,s)$. In this linearized expression, $\delta a \equiv a - \langle a\rangle$, $\alpha(L,s) = e^{i\theta|\alpha(0,s)|^2}\alpha(0,s)$, $\mu(s) = e^{i\theta|\alpha(0,s)|^2}(1 + i\theta|\alpha(0,s)|^2)$, and $\nu(s) = i\theta\alpha^2(0,s)e^{i\theta|\alpha(0,s)|^2}$. These expressions follow from Eqs. (S49), (S54) and (S55). The linearized approximation corresponds to dropping the terms that are quadratic in the fluctuation operators δa and $\delta a^\dagger$. Dropping those quadratic terms ensures that higher-order correlations do not couple to the mean (since $\langle \delta a\rangle$ and $\langle \delta a^\dagger\rangle$ are zero by construction). In what follows, we go beyond the linearized approximation by evaluating the contribution of the quadratic terms. Retaining those quadratic terms, $n_{\omega,\Delta\omega}$ is given by

$$n_{\omega,\Delta\omega} = \frac{\Delta\omega}{\pi} \left[\int ds ds' e^{-i\omega(s-s')} \text{sinc}\left(\Delta\omega(s-s')\right) \left(\alpha^*(L,s)\alpha(L,s') + \langle \delta a^\dagger(L,s)\delta a(L,s')\rangle\right)\right]. \tag{S69}$$

The second term depends on the statistics (specifically, the covariance) of the initial pulse. Let us first consider the case of an input coherent state. Then, one gets:

$$n_{\omega,\Delta\omega} = \frac{\Delta\omega}{\pi} \left[\int ds ds' e^{-i\omega(s-s')} \text{sinc}\left(\Delta\omega(s-s')\right) \left(\alpha^*(L,s)\alpha(L,s') + |\nu(s)|^2\delta(s-s')\right)\right]. \tag{S70}$$

To make considerations simpler, we consider a bandwidth $\Delta\omega \ll \tau^{-1}$ where τ is the pulse duration. In that case, the first term reduces to $|\alpha(L,\omega)|^2$. Therefore, the evaluation of the validity of the linearized approximation reduces to comparing $|\alpha(L,\omega)|^2$ and $\int ds|\nu(s)|^2$. To make clear the general physics, we give a general order of magnitude estimate for these two quantities. The term $|\alpha(L,\omega)|^2$ is simply the spectral density, and can be estimated as $|\alpha(L,\omega)|^2 \sim$ [number of photons per pulse]/[spectral bandwidth]. In ultrafast nonlinear optics in fibers, typical pulse photon numbers are $10^8 - 10^{10}$ (corresponding to $1 - 100$ mW scale average powers with a 0.1 GHz repetition rate). Let us consider the "worst-case scenario" in terms of the spectral bandwidth: a bandwidth on the order of the center frequency itself, so $\Delta\omega \sim \omega$. Then the average spectral density is on the order of $10^{-7} - 10^{-5}$. Meanwhile, the term $\int ds|\nu(s)|^2$ can be estimated as $\int ds|\nu(s)|^2 \sim$ [pulse duration] $\times$ [peak nonlinear phase]^2. Taking the nonlinear phase we assumed in the manuscript of 20π and a pulse duration on the order of 100 fs yields a number on the order of 10^{-10}. Therefore, in the shot-noise limited case, the relative magnitude of the correction is $10^{-3} - 10^{-5}$.

A related question is the effect of squeezing, which enhances noise in certain quadratures. Repeating the derivation shows that the contribution of the fluctuation term is just enhanced by $N(s)$ and $P(s)$. Taking the peak value of these quantities in Fig. 2 of roughly 10 enhances the corrections to $10^{-2} - 10^{-4}$.

We have shown that fluctuations, even squeezing-enhanced fluctuations, generally provide a weak correction to the mean. They will provide a similar relative correction to the variance terms. The way to see this is that if we consider the linearized expression for the fluctuations in a quantity such as the photon number $\delta(a^\dagger a) = \alpha\delta a^\dagger + \alpha^*\delta a + \delta a^\dagger \delta a$, the linearization approximation again amounts to dropping the quadratic term. If we wish to evaluate a variance for the case of Gaussian input statistics (such that odd-order moments vanish), then evaluating the magnitude of the correction again amounts to comparing a quantity like $\langle \delta a^\dagger \delta a \rangle$ with $|\alpha|^2$, which is precisely the same type of comparison we considered when looking at corrections to the mean.

We conclude this section by noting that the higher end of the relative correction should be considered as a close to worst-case scenario, as 10^8 is on the low side for many ultrafast nonlinear optics experiments in fibers, 20π is a fairly large phase shift, and a bandwidth of ω is a very large broadening. Nevertheless, even in this case, linearization represents a very good approximation.

S3.4. Other structures for squeezing and optimal noise control

The analysis of self-phase modulation by displaced squeezed vacuum in Section 3 is based on a delta-correlated structure for the Gaussian quantum correlations. Namely, due to the way we proposed to generate squeezed vacuum, the correlations were of the form $N(s, s') = N(s)\delta(s-s')$ and $P(s, s') = P(s)\delta(s - s')$. Nevertheless, there are many other types of correlations that could have been considered, and there is also a question of what input correlations lead to the *optimal* noise reduction.

In order to keep the scope of the manuscript contained, we discuss these points in the manuscript largely at a qualitative level. We will also discuss this from the standpoint of the single-mode squeezing case, where N and P become separable as a consequence of the Bloch-Messiah decomposition [6]. In particular, the input phase-insensitive correlations $N(s, s')$ can generally be expressed in the form $\sum\limits_{\lambda} \rho_\lambda \eta^*_\lambda(s)\eta_\lambda(s')$, while the phase-sensitive correlations can be expressed as $\sum\limits_{\lambda} \sigma_\lambda \eta_\lambda(s)\eta_\lambda(s')$, where the λ index is denoting squeezing "modes" called Schmidt modes. In this case, where we are describing correlations of a time-domain field, the resulting

modes $\eta(s)$ are normalized continuous-time functions which we refer to as squeezing modes. The quantities $\rho_\lambda = \sinh^2 r_\lambda$ and $\sigma_\lambda = \sinh r_\lambda \cosh r_\lambda$ quantify the degree of squeezing of each of the squeezing modes. In this case where one squeezing mode is dominant, we can approximate $N(s,s') = \rho\eta^*(s)\eta(s')$ and $P(s,s') = \sigma\eta(s)\eta(s')$. In this case, the noise of some quantity $(\Delta X)^2$ can be written as (following Eq. (6) of the main text):

$$(\Delta X)^2 = \int ds \left|\frac{\partial X_\omega}{\partial\alpha(0,s)}\right|^2 + 2\rho\left|\int ds\, \eta^*(s)\frac{\partial X}{\partial\alpha(0,s)}\right|^2 + 2\sigma \mathrm{Re}\left(\int ds\, \eta(s)\frac{\partial X}{\partial\alpha(0,s)}\right)^2.$$

As can be seen from this, the ability to reduce noise by injecting (Gaussian) quantum-correlated light depends on the overlap between the squeezing mode $\eta(s)$ and $\eta^*(s)$ and the gradient $\frac{\partial X}{\partial\alpha(0,s)}$ of the classical dynamics. Getting the maximum noise reduction in the single-mode squeezing case corresponds to optimizing over $\eta(s)$ subject to the constraint that it is normalized ($\int ds|\eta(s)|^2 = 1$). One thing that is clear from this expression is that a squeezing mode with a correlation time which is very short compared to the pulse duration of the "classical" or displaced part of the input pulse will not be very effective unless the squeezing magnitudes (encoded in ρ, σ) are high.

A longer correlation time could enable better performance. However, a qualitative difference from the delta-correlated case is that if $\eta(s)$ has a sign change, they will be sensitive to sign changes in the derivatives $\frac{\partial X}{\partial\alpha(0,s)}$. In other words, the optimal squeezing mode profile (i.e., that which maximizes the overlaps) depends specifically on the classical dynamics encoded in $\frac{\partial X}{\partial\alpha(0,s)}$. Therefore, while it may be simpler to control and characterize single-mode squeezing, the ability to perform noise-control is sufficiently non-trivial that we consider it as a subject for future study.

S3.5. Influence of temporal dispersion

In this section, we discuss the effect of finite temporal dispersion (in contrast to the main text where we considered an input pulse around the zero-dispersion wavelength). The impact of dispersion is complex, insofar as in many sources, light may propagate for several dispersion lengths $L_D \sim \tau^2/|\beta_2|$ where τ is the pulse duration and β_2 is the group velocity dispersion (with dimensions of $[\beta_2] = T^2/L$, with T time and L length). In this case, the spectrum will be very different compared to the case of zero dispersion – and it will change drastically depending on the sign of the dispersion as well. The advantage of considering the case of zero-dispersion is of course that it is an analytically solveable case. Further, it is also experimentally realizable via dispersion engineering especially in integrated photonic platforms such as lithographically etched

waveguides; see [7] for an example.

Moreover, cases with Kerr nonlinearity and dispersion tend to not be analytically solveable except in very special cases (such as solitons, which don't entail much spectral broadening). Generally, the effect of dispersion is to change the squeezed state that is needed to achieve noise reduction. The noise reduction will also generally be frequency-dependent, rather than being completely broadband. In any case, by optimizing the squeezing modes (see previous section), we expect that it will be possible to have strong control over noise of intensity and phase for frequencies of interest.

---

[1] Hans-A Bachor and Timothy C Ralph. *A guide to experiments in quantum optics*. John Wiley & Sons, 2019.

[2] Hermann A Haus. Waves and fields in optoelectronics. 1984.

[3] Marlan O Scully and M Suhail Zubairy. Quantum optics, 1999.

[4] Hermann A Haus. *Electromagnetic noise and quantum optical measurements*. Springer Science & Business Media, 2000.

[5] Govind P Agrawal. Nonlinear fiber optics. In *Nonlinear Science at the Dawn of the 21st Century*, pages 195–211. Springer, 2000.

[6] N Quesada, LG Helt, M Menotti, M Liscidini, and JE Sipe. Beyond photon pairs—nonlinear quantum photonics in the high-gain regime: a tutorial. *Advances in Optics and Photonics*, 14(3):291–403, 2022.

[7] Marc Jankowski, Carsten Langrock, Boris Desiatov, Alireza Marandi, Cheng Wang, Mian Zhang, Christopher R Phillips, Marko Lončar, and MM Fejer. Ultrabroadband nonlinear optics in nanophotonic periodically poled lithium niobate waveguides. *Optica*, 7(1):40–46, 2020.
